# Fermented olives (*Olea europaea* L.): A detailed insight into morphological changes and phenolic profile from harvest to jar

**DOI:** 10.1016/j.fochx.2025.102309

**Published:** 2025-02-21

**Authors:** Tea Burin, Mariana Cecilia Grohar, Jerneja Jakopic, Robert Veberic, Metka Hudina

**Affiliations:** University of Ljubljana, Biotechnical Faculty, Department of Agronomy, Jamnikarjeva 101, SI-1000 Ljubljana, Slovenia

**Keywords:** antioxidant capacity, brine, debittering, fermentation, oleuropein, pickling

## Abstract

The aim of this research was to determine the changes in fruit quality parameters, antioxidant capacity and phenolic content during natural fermentation of five olive cultivars. During processing, the fruit weight and firmness decreased and dry matter increased in all cultivars. Nine phenolic compounds were studied in detail during natural fermentation for the first time. 'Istrska belica' have the highest contents of phenolics, which require the longest processing period. Oleuropein content decreased during processing in all cultivars, resulting in fruit browning, a decrease in antioxidant capacity and an increase in other derived phenolic compounds. We found that the contents of bitter phenolic compounds stabilized a few months before the end of processing and the bitter taste in the final products did not differ between cultivars, although their content varied between cultivars. Understanding phenolic variations during the olive processing could reduce processing time and increase phenolic content in the final product.

## Highlights

• The oleuropein content at harvest affects the length of processing.
• Decrease in oleuropein causing fruit browning and decrease in antioxidant capacity.
• The content of bitter compounds did not change over the last few months of processing.
• The bitter taste of the final products did not differ between cultivars.
• Understanding the phenolic changing during processing could reduce processing time.

## 1 Introduction

Table olives (*Olea europea* L.) are a traditional product and an important component of the Mediterranean diet (Charoenprasert & Mitchell, 2012). They are a well-established functional food, mainly due to their high polyphenol content, with the predominant group of secoiridoides, which display anti-inflammatory, antioxidant, cardioprotective, neuroprotective and anticancer activities (Filardo et al., 2024; Tsantili, 2014). They also contribute to long shelf-life and organoleptic properties (Huang et al., 2024). The beneficial effects of table olive consumption are partly attributed to the phenolic content profile, which differs from olive oil (López-Salas et al., 2024; Savaş & Uylaşer, 2013). Among these compounds, oleuropein stands out as the primary phenolic compound in olive fruit, known for its notably bitter taste (Cui et al., 2021). For this reason, the fruit cannot be eaten fresh, but must undergo a series of processes that vary considerably among regions and cultivars (Ramírez et al., 2015).

Two procedures are commonly used for the industrial production of table olives: the chemical and the natural fermentation process (Durante et al., 2018). The chemical methods, used for Spanish-style green olives in brine and California-style black olives in brine, involves the hydrolyzation of oleuropein by treating the fruit with a diluted solution of sodium hydroxide (NaOH) (Malheiro et al., 2011), which breaks the ester bond of oleuropein, resulting in the formation of hydroxytyrosol and elenolic acid, both of which are non-bitter compounds (Vaccalluzzo et al., 2022). Although effective this method contributes to the pollution of the environment due to the waste it produces (Savaş & Uylaşer, 2013).

Conversely, in the natural fermentation process commercially used for Greek-style black olives, lactic acid bacteria and enzymes, present in the raw material, are responsible for the fermentation of table olives (Benincasa et al., 2015; Othman et al., 2009). During processing oleuropein is enzymatically hydrolyzed by *β-glucosidase* into glucose and oleuropein aglycone (Vaccalluzzo et al., 2022), being then hydrolyzed by *esterases* to elenolic acid and hydroxytyrosol (Fayek et al., 2021).

Slovenian Istria is the most important olive growing region in Slovenia. For pickling, producers in this region use mainly green olives, which are processed by natural spontaneous fermentation. The duration of the debittering process typically lasts between six and twelve months, which is longer that chemically processed methods (Valenčič et al., 2010). However, this method ensures the highest content of phenolic compounds and antioxidant capacity in the final product compared to other methods (Charoenprasert & Mitchell, 2012; Özcan & Uslu, 2023; Sahan et al., 2013). They also retain their crunchiness and have a higher sensory quality which make them a healthy snack option and do not have a negative impact on the environment compared to chemically processed olives (Ozturk, 2014).

Several studies have focused on variations in phenolic compounds during chemical processing methods (Ambra et al., 2017; Johnson et al., 2018; Özcan & Uslu, 2023; Sahan et al., 2013), and in their final products (Kazou et al., 2023; Malheiro et al., 2014). However, naturally fermented olives are rich in phenolic compounds, antioxidants and fibre, while being low in sugar (López-López et al., 2024), making them beneficial to human health. Unfortunately, detailed analysis of the changes in phenolic compounds during natural fermentation, especially in green olives, is still scarce. The main objective of the present work is to elucidate the mechanisms by which natural green olives lose their bitterness, specifically the variations in phenolic compounds content, antioxidant capacity and fruit quality parameters during natural spontaneous fermentation of 'Istrska belica', 'Štorta', 'Leccino', 'Mata', and 'Ascolana tenera'. Additionally, a sensory analysis of the final products was also conducted. Understanding the variations during the olive processing could optimize processing techniques, which contribute in the highest antioxidant and nutritional value and highest quality of the finished product, which can have a positive benefit on our well-being and also prolonged a shelf life of product.

## 2 Materials and methods

### 2.1 Olive fruits processing method

**Fig. 1:**
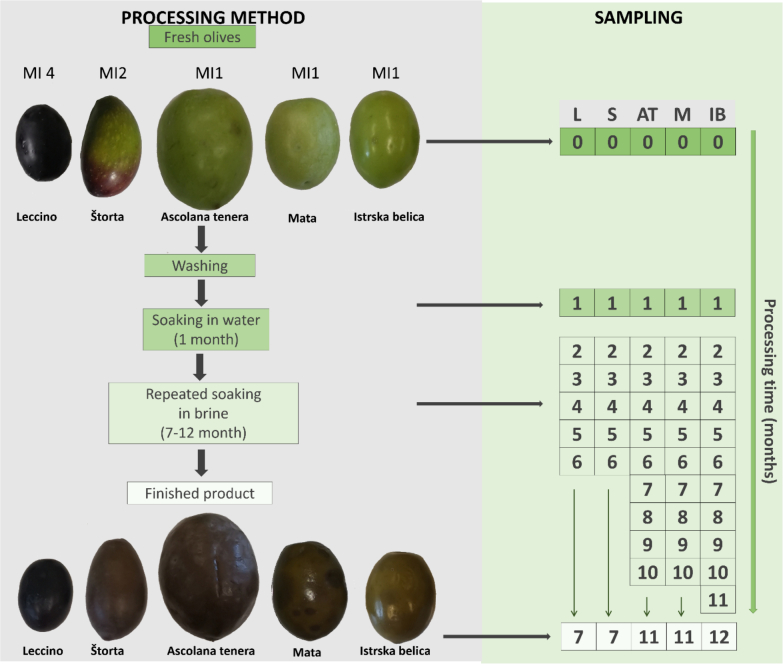
The processing method andsampling scheme of table olives cvs. 'Leccino' (L), 'Štorta' (S), 'Ascolana tenera' (AT), 'Mata' (M), and 'Istrska belica' (IB).

Olive fruits of the cultivars 'Istrska belica' (IB), 'Štorta' (S), 'Leccino' (L), 'Mata' (M), and 'Ascolana tenera' (AT) were collected one month before the expected harvest date for each cultivar, during the 2021 season in the olive orchard in Izola, Slovenian Istria. The cultivar 'Istrska belica', 'Mata' and 'Ascolana tenera' were harvest at maturity index (MI) 1, 'Štorta' at MI 2 and 'Leccino' at MI 4 (Fig. 1). The experiment was carried out on 40 kg of hand-picked and healthy fruit from each cultivar. After washing the fruit with tap water, they were placed in 100-litre barrels and left at room temperature (22-25 °C) to allow a natural fermentation. For the first thirty days, the fruits were soaked in tap water, which was changed every two days. At the end of this period, fruits were soaked in 7 % (w/v) NaCl brine and changed every five days. Olives were analyzed at harvest, after soaking in tap water, every month when fruits were soaking in brine, and at the end of process (Fig. 1) which was identified by a sequenced number (0-12). Each cultivar took a different time to debitter (Fig. 1). At each sampling date, fruits of each cultivar were evaluated in colour, firmness and weight then fruits were pitted, weighted, freeze-dried (CHRIST, Alpha 1-4 LSC basic, Germany), homogenized with an analytical mill (A11 basic, IKA, Germany) under liquid nitrogen, and stored at -20 °C until further analysis.

### 2.2 Physical measurements and maturity index (MI)

Fruit colour was measured by a colourimeter (CR-10 Chroma, Minolta, Osaka, Japan) and was expressed in lightness (L*), hue angle (*h°*) and chroma (C*). Colour measurements were made on the bottom of the fruit. Firmness was recorded on two sides on the equatorial zone at 90°, using a penetrometer model FT 01 (QA Supplies, Virginia, USA) with a cylindrical probe of 1.5 mm. The percentage of dry matter was calculated from the weighted fruits before and after freeze-drying. The maturity index (MI) was conducted on 100 fruits per maturity stage according to the method proposed by (Peragón, 2013) based on the evaluation of skin and pulp colour on a scale ranging from 0 to 7.

### 2.3 Extraction and determination of phenolic compounds

Extraction was performed as described in (Ivancic, Grohar, et al., 2022). 200 mg of the sample was poured over 3 mL of 100 % MeOH for each sample. After vortexing, the samples underwent a 60-minute treatment in a cooled ultrasonic bath (Sonis 4 ultrasonic bath; Iskra pio, Sentjernej, Slovenia) placed in ice water. Subsequently, the samples were centrifuged (5810 R; Eppendorf, Hamburg, Germany) at 10 000 rpm for 5 minutes at 4 °C, and the supernatant was filtered through 0.2 μm polyamide filters (Chromafil® AO-20/25; Macherey-Nagel, Düren, Germany) into vials.

Phenolic compounds were identified and quantified with UHPLC-MS system (Thermo Fisher Scientific, Waltham, MA, USA) with the column C18 (Phenomenex Gemini CA, USA; 150 × 4.6 mm, 3 μm). The experimental conditions on the mass spectrometer were previously outlined (Ivancic, Jakopic, et al., 2022). Phenolics were identified using literature data and by comparing their UV-Vis spectra and retention times with standards and confirmed with a mass spectrometer and with an electrospray interface (ESI) operating in negative ion mode (Table A.1). Compounds for which standards were not available were expressed as a similar standard: demethyloleuropein, oleuropein aglycone, elenolic acid, oleoside, ligstroside, ligstroside aglycone, and oleacein on oleuropein; hydroxytyrosol glucoside on hydroxytyrosol. All contents are expressed as mg/kg of dry weight (DW).

### 2.4 Antioxidant capacity

The free radical scavenging activity of the olives was measured using the DPPH (2,2-diphenyl-1-picrylhydrazyl) method. For analysis, 50 μL of the 25-times diluted extract for phenolic compound determination, and 200 μL of a 0.1 mM DPPH methanolic solution was mixed and allowed to react in the dark at room temperature for 20 min. The absorbance was determined at 517 nm using a microplate reader (BioTek synergy HTX Multimode Reader, Headquarters, US). 100 % MeOH (v/v) was used as a blank solution, and a DPPH solution with 50 μL of MeOH as a negative control. Trolox was used for standard calibration, and results obtained were expressed as mmol Trolox/100 g DW.*2.5 Sensory analysis*

The sensory attributes were assessed by eight evaluators, who rated the intensity of the basic flavors (saltiness, bitterness and sourness) and three textures (hardness, crunchiness and fibrousness) on the 8 cm line. The data were obtained by measuring the marks in cm which was considered as the score (Fig. 5., Table A.4). The evaluation sheet was taken from the International Olive Council (2011) appendix on sensory analysis of table olives. Ethical permission was not required to carry out the sensory evaluation.

### 2.5 Chemicals and standards

HPLC-grade methanol and formic acid for the extraction of the phenolics were purchased from Sigma-Aldrich (Steinheim, Germany). For the mobile phases, grade acetonitrile and formic acid from Fluka Chemie (Buch, Switzerland) were used. For antioxidant capacity 2,2-dipheny-1-picrylhydrazyl and Trolox from Sigma-Aldrich (Chemi GmbH, Steinheim, Germany) were used. The following standards were used for the quantification of phenolic compounds: Fluka Chemie (Buch, Switzerland): caffeic acid; Sigma-Aldrich (Chemi GmbH, Steinheim, Germany): 3-hydroxytyrosol and oleuropein; PhytoLab (Germany): oleoside-11-metyl ester and tyrosol; HWI group (Germany): verbascoside. The water for phenolic compounds extraction and mobile phases was double distilled and purified with a Milli-Q Millipore system (Merck Millipore, Billerica, MA, USA).

### 2.6 Data and statistical analysis

Statistical analysis was performed using the R-commander statistical software i386 4.3.0. Significant differences between sampling dates for each cultivar were examined using a one-way analysis of variance (ANOVA) with a Tukey's test at 95 % confidence. However, the results of the tasting analyses of the final product were compared between cultivars using the same tests. Data are expressed as mean ± standard deviation (SD). In order to determine the phenolic compounds that contribute the most to the data variability, we used principal component analyses (PCA). Due to the large number of data, only phenolic compounds with cos^2^ > 0.6 were included in the analyses (Fig. 4).

Hierarchical clustering of fruit colour during processing was made with Palentological Statistic software (version 4.17c), using Euclidean distance.

## 3 Results and discussion

### 3.1 Fruit quality parameters

During olive processing, fruit quality parameters were measured (Table 1). At harvest, the fruit weight was 6.96 g in 'Ascolana tenera', 4.55 g in 'Mata', 3.88 g in 'Štorta', 3.44 g in 'Istrska belica' and 3.23 g in 'Leccino'. During processing, it decreased in all cultivars, which was also observed before (Ozdemir et al., 2018). The weight loss during olive processing is the result of the movement of water from the fruit into the brine by diffusion due to the osmotic pressure (Ozturk, 2014). The percentage of dry matter at harvest also varied between cultivars and increased in all cultivars during the processing period, 'Leccino' by 8.8 %, 'Štorta' by 8.7 %, 'Ascolana tenera' by 6.6 %, 'Mata' by 8.6 % and 'Istrska belica' by 7.9 %, which was more pronounced than the decrease in fruit weight. Ozdemir et al. (2018) stated that during brining, not only the water, but also other organic compounds, such as phenolic compounds, sugars, vitamins and minerals leach out of the olive flesh, which could explain the difference between dry matter and fruit weight. Similar to other processing method (López-López et al., 2024; Ozdemir et al., 2018), all cultivars in our study showed a decrease in firmness during processing. This softening occurs because fermentation lowers the pH of the brine, which in combination with enzyme activity, leads to the degrade cell wall (Campus et al., 2018).

**Table 1:** Fruit weight (g), firmness (N) and dry matter (%) of cvs. 'Leccino', 'Štorta', 'Ascolana tenera', 'Mata' and 'Istrska belica' during olive processing (0-12).

	Processing time	'Leccino'	'Štorta'	'Ascolana tenera'	'Mata'	'Istrska belica'
Fruit weight (g)	0	3.23 ± 0.35 b	3.88 ± 0.47 b	6.96 ± 0.84 c	4.55 ± 0.50 b	3.44 ± 0.45 a
	1	3.18 ± 0.33 ab	3.72 ± 0.37 b	6.66 ± 0.99 bc	4.55 ± 0.84 b	3.34 ± 0.48 ac
	2	2.94 ± 0.20 ab	2.79 ± 0.52 a	6.69 ± 1.04 bc	4.53 ± 0.61 b	3.30 ± 0.14 ab
	3	3.09 ± 0.31 ab	3.04 ± 0.42 a	6.48 ± 0.74 ac	4.39 ± 0.38 ab	3.25 ± 0.35 ab
	4	2.89 ± 0.31 ab	3.04 ± 0.36 a	6.36 ± 1.06 ac	4.30 ± 0.43 ab	3.28 ± 0.50 ab
	5	2.95 ± 0.40 ab	3.10 ± 0.65 a	6.26 ± 0.83 ac	4.12 ± 0.61 ab	3.24 ± 0.34 ab
	6	2.93 ± 0.38 ab	2.85 ± 0.45 a	6.15 ± 1.14 ac	4.12 ± 0.64 ab	3.17 ± 0.28 ab
	7	2.83 ± 0.33 a	3.00 ± 0.34 a	6.09 ± 1.00 ac	3.89 ± 0.79 ab	3.13 ± 0.23 ab
	8			5.68 ± 0.97 ab	4.17 ± 0.71 ab	3.02 ± 0.35 bc
	9			5.60 ± 1.06 ab	3.91 ± 0.66 ab	3.05 ± 0.31 ab
	10			5.44 ± 0.91 a	3.71 ± 0.71 a	3.02 ± 0.24 bc
	11			5.41 ± 0.89 a	3.75 ± 0.46 a	2.99 ± 0.20 bc
	12					2.91 ± 0.14 b
Firmness (N)	0	1.06 ± 0.15 c	1.78 ± 0.59 a	1.11 ± 0.36 e	1.74 ± 0.58 e	1.99 ± 0.48 e
	1	0.95 ± 0.55 c	1.64 ± 0.57 ab	0.90 ± 0.5 ce	1.45 ± 0.46 de	1.32 ± 0.55 d
	2	0.73 ± 0.33 bc	1.66 ± 0.66 ab	0.90 ± 0.34 ce	1.23 ± 0.55 cd	1.12 ± 0.48 cd
	3	0.72 ± 0.28 bc	1.17 ± 0.65 ab	0.95 ± 0.17 de	1.16 ± 0.51 cd	0.96 ± 0.53 bd
	4	0.49 ± 0.20 ab	0.72 ± 0.24 bc	0.84 ± 0.29 ce	1.01 ± 0.45 bd	0.98 ± 0.63 bd
	5	0.48 ± 0.33 ab	0.90 ± 0.51 cb	0.76 ± 0.23 bcd	0.92 ± 0.46 abc	0.77 ± 0.34 abc
	6	0.46 ± 0.19 ab	0.70 ± 0.29 cb	0.64 ± 0.17 ac	0.77 ± 0.18 abc	0.79 ± 0.41 abc
	7	0.28 ± 0.11 a	0.60 ± 0.20 d	0.55 ± 0.20 ab	0.74 ± 0.39 abc	0.79 ± 0.43 abc
	8			0.45 ± 0.11 a	0.79 ± 0.33 abc	0.75 ± 0.38 abc
	9			0.47 ± 0.13 a	0.65 ± 0.37 ab	0.55 ± 0.23 ab
	10			0.38 ± 0.14 a	0.60 ± 0.21 ab	0.47 ± 0.26 ab
	11			0.41 ± 0.16 a	0.46 ± 0.26 a	0.59 ± 0.32 ab
	12					0.41 ± 0.19 a
Dry matter (%)	0	43.17 ± 0.36 a	43.19 ± 3.24 a	32.06 ± 0.47 a	31.31 ± 1.44 a	44.32 ± 0.97 a
	1	43.96 ± 0.38 a	39.85 ± 1.08 a	34.50 ± 1.40 abc	36.27 ± 0.94 ab	44.65 ± 3.57 a
	2	49.98 ± 6.10 ab	46.49 ± 3.61 ab	34.64 ± 0.17 abc	36.95 ± 0.67 ab	47.28 ± 2.13 ab
	3	50.44 ± 1.82 ab	45.65 ± 3.98 ab	33.76 ± 0.74 ac	36.04 ± 1.17 ab	47.84 ± 0.14 ab
	4	50.16 ± 3.15 ab	45.81 ± 2.85 ab	35.24 ± 1.65 ad	35.98 ± 1.16 ab	47.03 ± 1.32 ab
	5	49.40 ± 0.77 ab	44.15 ± 3.26 ab	36.43 ± 1.63 cd	35.63 ± 1.90 ac	49.76 ± 0.15 ab
	6	48.54 ± 1.60 ab	45.69 ± 0.75 ab	37.80 ± 1.14 bd	41.75 ± 0.86 b	48.62 ± 0.14 ab
	7	51.99 ± 2.03 b	51.89 ± 1.83 b	36.85 ± 0.62 cd	41.72 ± 3.71 b	47.52 ± 1.02 ab
	8			37.10 ± 1.18 cd	39.60 ± 2.1 bc	50.42 ± 2.79 b
	9			35.70 ± 1.69 cd	39.96 ± 1.92 bc	50.79 ± 1.56 b
	10			38.29 ± 1.31 d	38.56 ± 3.81 bc	51.49 ± 0.35 b
	11			38.61 ± 0.39 d	39.87 ± 0.73 bc	51.28 ± 3.83 b
	12					52.15 ± 1.14 b

The values are means ± SD. Different letters indicate statistical differences among different month of processing according to Tukey’s multiple range test (*p* ≤ 0.05).

**Fig. 2:**
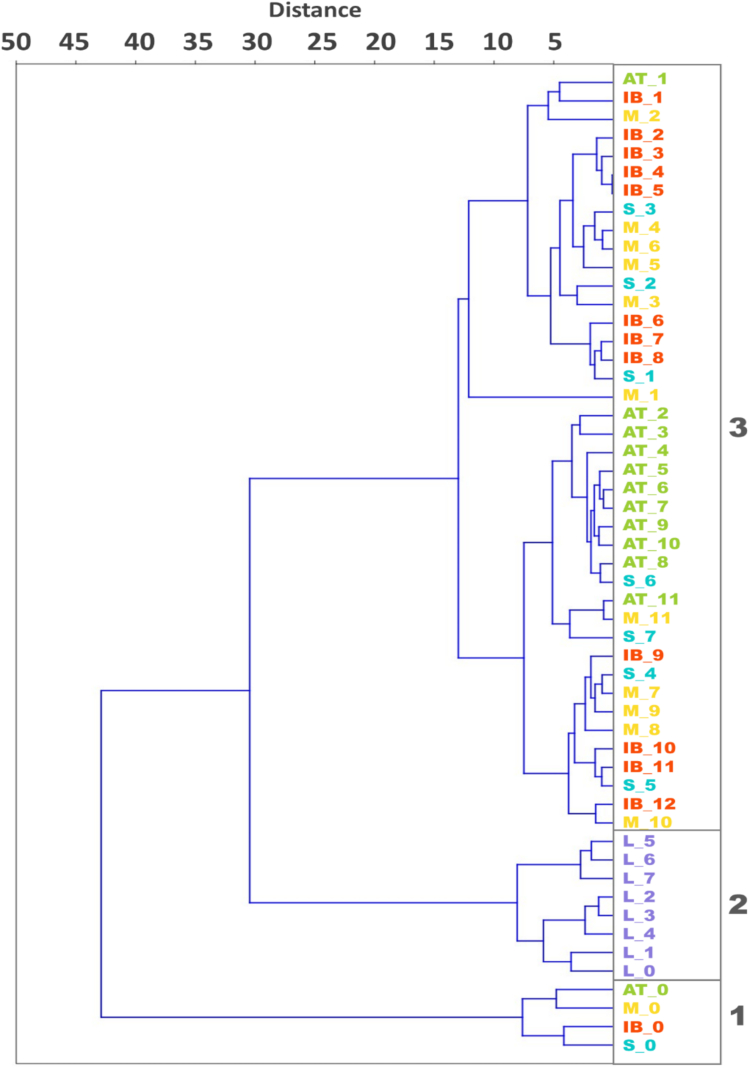
Hierarchical clustering of the colour of cvs. 'Leccino' (L), 'Štorta' (S), 'Ascolana tenera' (AT), 'Mata' (M) and 'Istrska belica' (IB) olives during processing (0-12).

It is known that during processing, fruit colour changes due to the conversion of their pigments (López-López et al., 2024). Fruit colour results were grouped by hierarchical clustering (Fig. 2, Table A.2.) in three groups. The first group includes all green cultivars ('Štorta', 'Ascolana tenera', 'Mata' and 'Istrska belica') at harvest, which are in other sampling dates clustered together in the third group. During the processing, their colour changes from intense green to grey brownish (Fig. 1, Fig. 2), which is the result of chlorophyll degradation and enzymatic oxidation of oleuropein and hydroxytyrosol (Ramírez et al., 2015). The second group includes data of all sampling dates of 'Leccino', which is the only cultivar with dark purple colour of fruits at harvest time, which showed minimal changes through the processing method.

### 3.2 Antioxidant capacity

Antioxidant capacity is an important aspect of olive processing, as it affects the quality and shelf life of the final product (Malheiro et al., 2011). Our results showed a statistically significant decrease in antioxidant capacity during the process in all cultivars (Table 2), which were also found in other cultivars (Othman et al., 2009; Sahan et al., 2013). The greatest decrease was observed in 'Štorta' and 'Leccino' (76.7 %), which underwent the shortest debittering process, following by 'Mata' (63 %), 'Ascolana tenera' (61 %) and 'Istrska belica' (47.8 %), which underwent the longest processing time. This decrease in antioxidant capacity is closely related to the reduction in phenolic compounds, which have been affected by enzymatic oxidation and microbial activity during processing, as well as the effectiveness of the overall debittering process (Campus et al., 2018; Charoenprasert & Mitchell, 2012).

**Table 2:** Antioxidant capacity of cvs. 'Leccino', 'Štorta', 'Ascolana tenera', 'Mata' and 'Istrska belica' during the olive processing (0-12).

Processing period	Leccino	Štorta	Ascolana tenera	Mata	Istrska belica
1	71.52 ± 3.98 a	33.87 ± 3.09 a	64.83 ± 2.31 a	77.23 ± 3.73 a	54.01 ± 0.88 a
2	46.20 ± 1.31 b	24.35 ± 0.82 b	61.85 ± 3.18 ae	66.63 ± 1.74 ae	54.43 ± 4.16 a
3	35.58 ± 1.16 cd	17.61 ± 1.00 c	60.36 ± 4.32 ae	60.39 ± 1.54 ef	51.41 ± 2.27 a
4	40.63 ± 3.61 bc	18.46 ± 1.95 c	60.52 ± 3.44 ae	49.45 ± 0.40 fgh	49.23 ± 5.94 ae
5	33.20 ± 1.68 de	12.14 ± 1.41 d	58.87 ± 0.97 ae	50.76 ± 0.25 fgh	48.36 ± 7.08 af
6	38.61 ± 2.53 ce	10.78 ± 2.39 d	54.33 ± 1.68 ef	55.47 ± 2.41 eg	47.37 ± 3.11 adf
7	28.40 ± 2.94 d	9.71 ± 1.28 d	47.44 ± 2.12 cf	47.46 ± 8.14 bgh	46.53 ± 0.65 abf
8	20.45 ± 1.43 f	7.89 ± 0.26 d	48.83 ± 6.59 bf	43.35 ± 4.32 dg	36.24 ± 4.82 cf
9			39.84 ± 1.93 bc	41.78 ± 1.96 dh	37.30 ± 5.04 cef
10			39.65 ± 2.58 bc	36.63 ± 2.10 bcd	34.62 ± 6.95 bc
11			38.48 ± 2.74 c	34.12 ± 0.89 cd	34.90 ± 3.97 bcd
12			24.97 ± 3.34 d	28.73 ± 0.52 c	32.65 ± 1.93 c
13					28.18 ± 1.61 c

The values are means ± SD. Different letters indicate statistical differences among different month of processing according to Tukey’s multiple range test (*p* ≤ 0.05).

### 3.3 Individual phenolic compounds

**Fig. 3:**
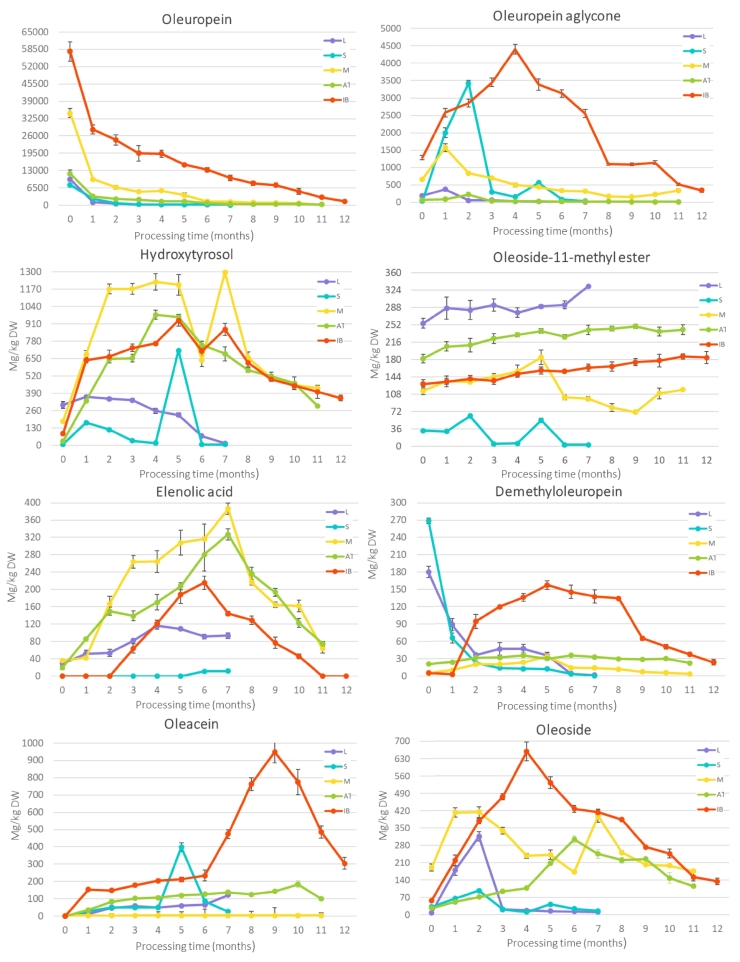

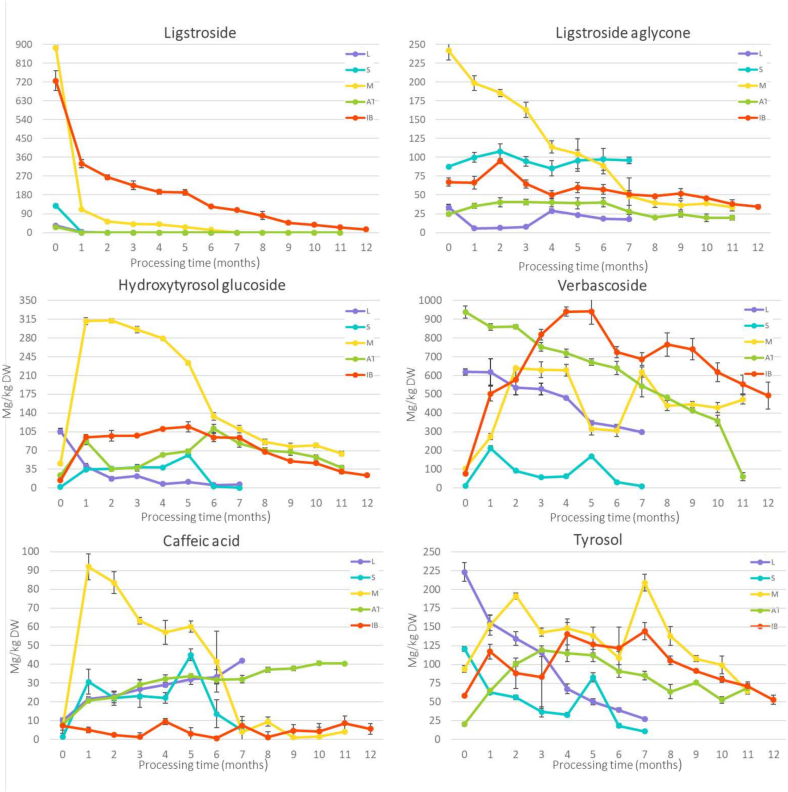
Changes in individual phenolic compounds during processing (from 0 to 12 months) method for cvs. 'Leccino' (L), ' Štorta' (S), 'Mata' (M), 'Ascolana tenera' (AT) and 'Istrska belica' (IB). The results are expressed as mg/kg dry weight (DW), with their standard deviations.

The phenolic profile of the olives was closely monitored during processing, focusing on 14 individual phenolic compounds in five cultivars (Fig. 3, Table A.3).These compounds included eight secoiridoids (oleuropein, oleuropein aglycone, oleoside-11-methyl ester, demethyloleuropein, elenolic acid, oleoside, ligstroside, oleacein, and ligstroside aglycone), two simple phenols (hydroxytyrosol and tyrosol), two phenylpropanoid glucosides (verbascoside, hydroxytyrosol glucosides) and one hydroxycinnamic acid (caffeic acid).

The phenolic profile of olive fruits, is influenced by various factors, including processing method, and type of fermentation (Conte et al., 2020; Malheiro et al., 2014, Malheiro, Sousa, Casal, Bento and Pereira, 2011). In these study **oleuropein** was the most abundant phenolic compound identified in all cultivars and its content was the highest at harvest in all cultivars. As noted by several other researches, the content of oleuropein in fresh olives predominately depend of genetic factors and the maturity stage of fruit (Charoenprasert & Mitchell, 2012; Kara, 2023). In our study, a significant decrease in oleuropein content was already evident after one month of processing for all cultivars, and over more until the end of processing. Oleuropein is known to be water-soluble compound (Ozturk, 2014) and is extracted from the pulp by osmotic pressure and diffusion when olives are soaked in water or brine during processing (Ozdemir et al., 2018; Romero, Brenes, Yousfi, et al., 2004). During this process, fermentation occurs mainly due to the activity of lactic acid bacteria *Lactobacillus plantarum*, with enzymes playing a crucial role in the degradation of oleuropein and other phenolic compounds (Benincasa et al., 2015; Pistarino et al., 2013). At the end of the process, the lowest content of oleuropein was observed in 'Leccino' (33 mg/kg DW), followed by 'Štorta' (98 mg/kg DW), 'Mata' (191 mg/kg DW), 'Ascolana tenera' (376 mg/kg DW) and 'Istrska belica' (1520 mg/kg DW). The decrease in oleuropein content was also observed during the debittering process of Spanish-style and Californian-style process in different cultivars (Özcan & Uslu, 2023; Sahan et al., 2013). 'Istrska belica' required the longest time to be processed, probably due to its high oleuropein content, which has antimicrobial properties, especially against the lactic acid bacteria responsible for its degradation, which can delay the process (Charoenprasert & Mitchell, 2012; Ozturk, 2014). Moreover, Blekas et al. (2002) reported that *L. plantarum* strains are grown when the total phenol content of the olives is relatively low and the fermentation brine contains no more than 8 % salt. From the results of all cultivars it can be seen that the oleuropein content become stable a few months before the end of processing. This may suggest that fermentation could be stopped earlier, but further research with detailed sensory analysis during processing should be carried out.

The decrease in phenolic compounds, especially of oleuropein content, during processing is also reflected in the decrease in antioxidant capacity (Ozturk, 2014; Sahan et al., 2013). However, the decrease in oleuropein content in olive fruits can also contribute to browning (Ramírez et al., 2015). As oleuropein is degraded by *β*-*glucosidases* and *esterases*, hydroxytyrosol is released and subsequently oxidized by polyphenol oxidase, which leads to browning (Charoenprasert & Mitchell, 2012). Our results of oleuropein content changes and antioxidation capacity also contribute this statement.

Oleuropein is hydrolyzed by *β-glucosidases* to non-bitter glucose and bitter **oleuropein aglycone** (Johnson et al., 2018; Vaccalluzzo et al., 2022; Volk et al., 2019). Our study is the first to report detailed variations in its content during the whole natural fermentation processing. At the beginning of the processing, the content of oleuropein glucoside increased in all cultivars, probably due to oleuropein degradation, until it reached its maximum (in 'Leccino' and 'Mata' after one month, in 'Štorta' and 'Ascolana tenera' after two months and in 'Istrska belica' after five months), after which it began to decrease. The decrease in oleuropein aglycon occurred at the same time when its degradation outpaced that of oleuropein, which can be partly observed by the increase in elenolic acid, one of its degradation products. Other authors reported about constant decrease in oleuropein aglycon after NaOH treatment (Ambra et al., 2017; Charoenprasert & Mitchell, 2012). However, the oleuropein aglycon content did not change statistically in the last two months of processing in 'Istrska belica', 'Štorta' and 'Mata', in the last four in 'Leccino', and nine in 'Ascolana tenera', which may suggest that fermentation could be stopped earlier.

Oleuropein aglycone is furtherly hydrolysed by *esterases* to non-bitter elenolic acid and **hydroxytyrosol** (Vaccalluzzo et al., 2022). Its content at harvest was 303 mg/kg DW in 'Leccino', 179 mg/kg DW in 'Mata', 87 mg/kg DW in 'Istrska belica', 30 mg/kg DW in 'Ascolana tenera', and 7 mg/kg DW in 'Štorta'. Hydroxytyrosol was the second most abundant phenolic compound in 'Ascolana tenera' (297 mg/kg DW), and the third in 'Mata' (429 mg/kg DW) and 'Istrska belica' (354 mg/kg DW). In 'Štorta' and 'Leccino' its content was 7 mg/kg DW and 15 mg/kg DW, respectively. Furthermore, hydroxytyrosol was found to be the most abundant phenolic compound in green table olives wastewaters, lye washing waste- water and brine (Kiai & Hafidi, 2014; Morelló et al., 2004). Our results show that, similar to oleuropein aglycone, the hydroxytyrosol content first increased and then decreased in the cultivars 'Ascolana tenera' and 'Leccino'. Interestingly, two peaks of hidroxytyrosol content were observed in 'Istrska belica', 'Štorta' and 'Mata', which aligns with finding using California-style and Greek-style processing methods (Ambra et al., 2017; Mastralexi et al., 2019; Özcan & Uslu, 2023). The initial increase in hydroxytyrosol content during processing is a result of oleuropein degradation, a pattern confirmed in some cultivars where hidroxytyrosol continued to increase even after oleuropein aglycone content started to decrease. However, the presence of two peaks of hydroxytyrosol during processing suggests that enzymatic activity might play a role, so further studies on the enzymes responsible for its degradation are needed.

**Oleoside-11-methyl ester** is formed by the degradation of oleuropein, by *esterases* (Johnson et al., 2018; Vaccalluzzo et al., 2022). At harvest, its content was the highest in 'Leccino' (256 mg/kg DW) and 'Ascolana tenera' (182 mg/kg DW), followed by 'Istrska belica' (129 mg/kg DW), 'Mata' (115 mg/kg DW), and 'Štorta' (32 mg/kg DW). Previous studies have reported that oleoside-11-methyl ester is present in higher content in lye-treated olives compared to fresh olives, with its content increasing as oleuropein decreases (Charoenprasert & Mitchell, 2012; Mastralexi et al., 2019; Sahan et al., 2013), which is consistent with our results. In 'Istrska belica', 'Ascolana tenera' and 'Leccino' content of oleoside-11-methyl ester increased during natural spontaneous fermentation and reached 184 mg/kg DW, 242 mg/kg DW and 333 mg/kg DW, respectively, by the end of processing. However, in 'Mata', the content increased until five months of processing and then decreased, ultimately reached a content comparable to that at harvest (118 mg/kg DW). Mastralexi et al. (2019) reported that the content of oleoside-11-methyl ester was below the detection threshold in fresh fruits, increased drastically immediately after NaOH treatment and then decreased to zero. In 'Štorta', the content of oleoside-11-methyl ester at the end of processing was lower than in fresh fruit (3 mg/kg DW). This decrease could be attributed to the increase in elenolic acid, which began to increase after five months of processing, coinciding with the decrease inoleoside-11-methyl ester. A similar decrease in oleoside-11- methyl ester has been reported in Spanish-style and Greek-style processing methods, because its glucose serves as a substrate for lactic acid bacteria during fermentation (Charoenprasert & Mitchell, 2012). Our results indicate that the variations in oleoside-11-methyl ester content are cultivar-specific. Notably, cultivars with lower oleoside-11-methyl ester content at harvest showed either no change or a further decrease in its content by the end of processing, while cultivars with higher initial content exhibited an increased during processing.

**Elenolic acid** can be synthesised from oleuropein aglycone by *esterases* or from oleoside-11-methyl ester by *β-glucosidases* (Vaccalluzzo et al., 2022). At harvest, the its content was the highest in 'Mata' (35 mg/kg DW), followed by 'Leccino' (29 mg/kg DW) and 'Ascolana tenera' (19 mg/kg DW), while in 'Istrska belica' and 'Štorta', it was too low to be detected. Like other degradation products of oleuropein, elenolic acid increases as the fruit ripens (Ozdemir et al., 2018). In our study, elenolic acid content increased in all cultivars at the beginning of processing, reaching its maximum before subsequently decreasing. The degradation of oleoside-11- methyl ester releases glucose, which serves as a substrate for lactic acid bacteria (Charoenprasert & Mitchell, 2012). However, the simultaneous accumulation of elenolic acid inhibits microbial activity involved in the lactic fermentation of olives (Ozdemir et al., 2018), which could explain its initial increase followed by a decrease. Additionally, elenolic acid is a relatively unstable and degrades under acidic conditions, which intensify as fermentation progresses (Ozdemir et al., 2018). At the end of the process, its content was 12 mg/kg DW in 'Štorta', 94 mg/kg DW in 'Leccino', 75 mg/kg DW in 'Ascolana tenera', 64 mg/kg DW in 'Mata' and 0 mg/kg DW in 'Istrska belica'. Despite its potential role in olive fermentation, elenolic acid has been poorly studied, probably due to its low concentrations at the beginning and end of processing, which makes it difficult to analyse.

**Demethyloleuropein** is produced by demethylation of oleuropein (Vaccalluzzo et al., 2022; Volk et al., 2019). At harvest, its content was the highest in 'Štorta' (269 mg/kg DW), followed by 'Leccino' (180 mg/kg DW), 'Ascolana tenera' (21 mg/kg DW), 'Istrska belica' (15 mg/kg DW) and 'Mata' (5 mg/kg DW), which could be related to their maturity, as more mature olives tend to have a higher demethyloleuropein content (Alagna et al., 2012). However, its variations during olive processing have not been well investigated, so our study is the first one that explore its changes during natural fermentation. Our results showed that demethyloleuropein decreased strongly in 'Štorta' and 'Leccino', which aligns with its knowninstability and rapid degradation after synthesis. One of its degradation product, oleacein (Johnson & Mitchell, 2018), increased at the beginning of processing, further supporting this observation. Interestingly, in 'Istrska belica' demethyloleuropein content increased until the fifth month of processing before returning to approximately its initial content in the final product. This trend is in good agreement with other authors (Ambra et al., 2017), in NaOH treated olives, suggesting that thedegradation dynamics of demethyloleuropein may depend on processing conditions. The observed increase may result from a faster degradation rate of oleuropein compared ti demethyloleuropein. In contrast, 'Ascolana tenera' and 'Mata' showed only minor fluctuations in demethyloleuropein content during processing. At the end of fermentation, the demethyloleuropein content were reduced to 0 mg/kg DW for 'Štorta', 2 mg/kg DW for 'Leccino', 3 mg/kg DW for 'Mata', 22 mg/kg DW for 'Ascolana tenera' and 24 mg/kg DW for 'Istrska belica'. These findings indicate that demethyloleuropein degradation is cultivar-specific, highlighting the need for further research to better understand the enzymatic and microbial mechanisms governing its degradation during fermentation.

Decarboxylation of the oleuropein aglycones results in the synthesis of bitter **oleacein** (Johnson & Mitchell, 2018), which can also be produced from demethyloleuropein (Volk et al., 2019). Oleacein is one of the most abundant secoiridoids in extra virgin olive oil, but its content is low in fresh fruit (Huang et al., 2024; López-Salas et al., 2024). In our study, the oleacein content was not detected in 'Štorta', 'Ascolana tenera' and 'Istrska belica’, while small amounts were present in 'Leccino' (4 mg/kg DW) and 'Mata' (5 mg/kg DW). To date, its content in processed olives has only been quantified in Greek-style olives by (Johnson et al., 2018), and no studies have monitored its variation during processing. Our results show that the oleacein content generally increased to a peak before decreased towards the end of the fermentation, except 'Mata', where its content remained stable throughout processing. The most pronounced increase was observed in cultivar 'Istrska belica', where oleacein reached 949 mg/kg DW after nine months before decrease. This could be attributed to the decrease in demethyloleuropein and oleuropein aglycon content, which became particularly evident in 'Istrska belica' after five months of processing.

**Oleoside**, known for its pharmacological propertied (Volk et al., 2019), is obtained by the hydrolysis of oleoside 11-methyl ester (Volk et al., 2019). At harvest, its highest content was in 'Mata' (190 mg/kg DW), followed by 'Istrska belica' (57 mg/kg DW), 'Štorta' (32 mg/kg DW), 'Ascolana tenera' (26 mg/kg DW) and 'Leccino' (8 mg/kg DW). While oleoside contentis known to increase during ripening (Ivancic, Jakopic, et al., 2022), its behaviour during natural fermentation is still unknown. In our study, oleoside content increased in most cultivars early in processing, probably due to enzymatic hydrolysis of oleoside-11-methyl ester, polloed by a decrease towards the end of processing. A similar trend was reported by Ambra et al. (2017), after NaOH treatment, suggesting that fermentation and alkalic treatments may have a comparable effect on oleoside stability By the end of the processing, the oleoside content was 176 mg/kg DW in 'Mata', 135 mg/kg DW in 'Istrska belica', 115 mg/kg DW in 'Ascolana tenera', 16 mg/kg DW in 'Štorta' and 11 mg/kg DW in 'Leccino'.

Ripe olives contain high levels of bitter phenolic compounds, including **ligstroside** that make the fruit inedible (Johnson & Mitchell, 2018). It accumulates in the pulp and skin during ripening as a protective mechanism against insects, pathogens and herbivores (Johnson et al., 2018). However, its fate during natural fermentation has not been well studied. In our study, ligstroside content was the highest in'Mata' (884 mg/kg DW) and 'Istrska belica' (726 mg/kg DW), while lower content found in 'Štorta' (128 mg/kg DW), 'Leccino' (34 mg/kg DW) and 'Ascolana tenera' (26 mg/kg DW). A significant decrease was observed in all cultivars, with the most rapid in the first month. Ligstroside was completely degraded in 'Štorta' and 'Ascolana tenera' early in the process, fell below the detection in 'Leccino‘after two months, and in 'Mata' after seven months of processing, similar to reported in California-style processed olives (Johnson et al., 2018). In contrast, 'Istrska belica' retained a low content (15 mg/kg DW) in the final product . Ambra et al. (2017) also found similar contents in cv. 'Nocellara del Belice' processed by the Spanish-style method. The reason for its decrease is probably due to *β-glucosidase* activity, which hydrolyses it into the ligstroside aglycone (Johnson et al., 2018; Kazou et al., 2023; Volk et al., 2019). This transformation was particularly evident in 'Ascolana tenera' and 'Štorta', where the aglycone content increased in parallel with ligstroside degradation. The rate and extent of ligstroside degradation is likely to depend on cultivar-specific enzymatic activity and microbial fermentation dynamics, explaining the differences observed between cultivars.

**Ligstroside aglycone** is a bitter compound (Johnson & Mitchell, 2018), that differ from oleuropein aglycone in one OH group (Johnson et al., 2018). This study is the first, which analyse its changes during processing. At harvest its content was most abundant in 'Mata' (242 mg/kg DW), followed by 'Štorta' (88mg/kg DW), 'Istrska belica' (67 mg/kg DW), 'Leccino' (34 mg/kg DW) and 'Ascolana tenera' (24 mg/kg DW). Despite the conversion of ligstroside to ligstroside aglycone, its highest content was still observed at harvest. This suggest that, rather than accumulating, ligstroside aglycone undergoes further transformations during fermentation. Johnson and Mitchell (2018) reported that ligstroside aglycone is further esterified to oleacein and/or elenolic acid, both of which increased at certain time points in our study., The decrease in ligstroside aglycone content in most cultivars indicates its rapidly degraded into further products. This was particularly evident in 'Mata', where its content sharply decreased over the seven months, coinciding with a strong increase in elenolic acid. In contrast, 'Štorta' remained stable contents of both elenolic acid and ligstroside aglycone throughout fermentation, suggesting limited enzymatic activity or conversion in this cultivar . In the final product, the content of ligstroside aglycon was 96 mg/kg DW in 'Štorta', 34 mg/kg DW in 'Mata' and 'Istrska belica', 20 mg/kg DW in 'Ascolana tenera' and 18 mg/kg DW in 'Leccino'. In all cultivars, no statistical changes were observed in the final months of fermentation, mirroring the trend seen with oleuropein. **Hydroxytyrosol glucoside**, a glycosylated derivative of hydroxytyrosol, enchances its **stability**, **solubility**, and **bioavailability** **(**Kiai & Hafidi, 2014). This study is the first to examine its variation during processing. At harvest, hydroxytyrosol glucoside content were harvest in 'Leccino' (106 mg/kg DW), followed by 'Mata' (46 mg/kg DW), 'Istrska belica' (14 mg/kg DW), 'Ascolana tenera' (14 mg/kg DW) and 'Štorta' (12 mg/kg DW). During processing, its content decreased only in 'Leccino', while in all other cultivars, it increased after the first month of processing and then decreased. This early increase, coinciding with fruit exposure to tap water, may result from oleuropein degradation, as previously reported (Romero, Brenes, García, et al., 2004). The subsequent decrease in hydroxytyrosol glucoside content in later processing stages likely reflects sugar cleavage, leading to a secondary increase in hydroxytyrosol. In addition, hydroxytyrosol content may rise due to enzymatic hydrolysis and diffusion into the brine (Kiai et al., 2020; Romero, Brenes, García, et al., 2004). In the final product, in 'Ascolana tenera', 'Istrska belica' and 'Mata' showed no differences in hydroxytyrosol glucoside content compared to the initial contents, whereas its content was lower in 'Leccino' (7 mg/kg DW) and 'Štorta' (1 mg/kg DW). These findings suggest that cultivar-specific factors influence the balance between glycosylation and hydrolysis during fermentation.

At harvest, **verbascoside**, together with oleuropein, was the most abundant phenolic compound in 'Ascolana tenera' (939 mg/kg DW) and 'Leccino' (619 mg/kg DW), which is in good agreement with other authors (Sahan et al., 2013). However, its content was much lower in 'Štorta' (12 mg/kg DW), 'Istrska belica' (78 mg/kg DW) and 'Mata' (104 mg/kg DW), indicating that verbascoside content is cultivar-specific. In 'Leccino' and 'Ascolana tenera', its content decreased during processing, while in 'Štorta', 'Istrska belica' and 'Mata' it initially increased to a maximum and then decreased. The increase of its content in brine during fermentation of green table olives with starter culture was observed by Benincasa et al. (2015), which could explain the variation during the first four months of green cultivars. In addition, Sahan et al. (2013) suggested that hydroxytyrosol could be converted into verbascoside, which may account for the observed relationship between hydroxytyrosol and verbascoside during processing. At the end of the processing, the verbascoside content varied greatly, in 'Štorta' (9 mg/kg DW), 'Leccino' (298 mg/kg DW), 'Ascolana tenera' (60 mg/kg DW), 'Istrska belica' (493 mg/kg DW) and 'Mata' (472 mg/kg DW). Interestingly, Morelló et al. (2004) found that verbascoside was only present in olives processed by natural fermentation, while in other chemically processed olives, its content could not be detected. Since detailed analysis of verbascoside during the process, has not been conducted, further studies investigating gene expression or enzyme activity would be valuable to better understand our results.

**Caffeic acid** is obtained from verbascoside by its hydrolysis (Othman et al., 2009). While some authors reported that caffeic acid is not present in fresh fruits (Kiai & Hafidi, 2014), this contradicts the finding of other authors (Ivancic, Jakopic, et al., 2022; Othman et al., 2009) and ours. At harvest, the highest caffeic acid content was found in 'Mata' (48 mg/kg DW) followed by 'Istrska belica' (17 mg/kg DW), 'Leccino' (10 mg/kg DW), 'Ascolana tenera' (8 mg/kg DW) and 'Štorta' (2 mg/kg DW). After one month of processing, its content increased in most cultivars. A constant increase during processing was observed in 'Leccino' and 'Ascolana tenera', which could be a consequence of the decrease of verbascoside. Other authors (Benincasa et al., 2015; Kara, 2023; Kiai et al., 2020) also observed an increase of caffeic acid during different processing methods . In 'Štorta', 'Istrska belica' and 'Mata', two major peaks in caffeic acid can be observed, probably due to the huge increase of verbascoside in the same period. The content of caffeic acid in the final product was 42 mg/kg DW in 'Leccino', 40 mg/kg DW in 'Ascolana tenera', 6 mg/kg DW in 'Istrska belica' and 4 mg/kg DW in 'Mata'.

**Tyrosol** is a hydrolysis product of ligstroside (Charoenprasert & Mitchell, 2012), and it is later converted to hydroxytyrosol (Bouallagui & Sayadi, 2018). At harvest, its content was the highest in 'Leccino' (223 mg/kg DW) and followed by 'Štorta' (121 mg/kg DW) and it decreased during processing. Science ligstroside content was high at harvest, of the decrease in tyrosol is likely due to the total degradation of its precursor. At harvest, the content of tyrosol in 'Ascolana tenera', 'Istrska belica' and 'Mata' was 20 mg/kg DW, 58 mg/kg DW and 94 mg/kg DW, respectively. Its content increased at the beginning of processing, probably due to ligstroside degradation, then varied differently across cultivars, ending in a lower content than at harvest. At the end of processing the highest content of tyrosol was found in 'Ascolana tenera' (69 mg/kg DW), followed by 'Mata' (65 mg/kg DW), 'Istrska belica' (53 mg/kg DW), 'Leccino' (27 mg/kg DW) and 'Štorta' (11 mg/kg DW). However, Sahan et al. (2013) reported an increase in tyrosol content during Spanish-style and Greek-style of processing. The variations in our results can be explained by the study of Kazou et al. (2023), who reported that different processing methods lead to different final tyrosol content.

**Fig. 4:**
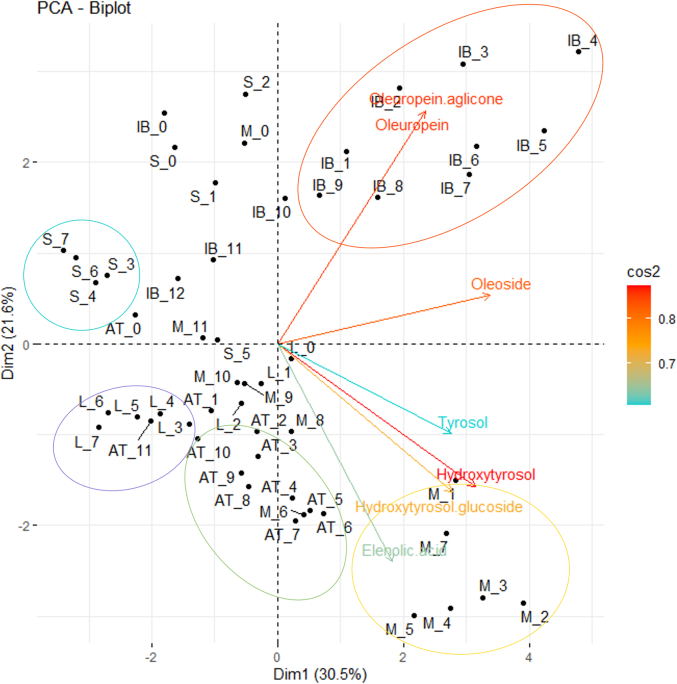
Score and loading bi-plot obtained with Principal Component Analysis for all phenolic compounds studied during processing (from 0 to 12 months) for cvs. 'Leccino' (L), 'Štorta' (S), 'Mata' (M), 'Ascolana tenera' (AT) and 'Istrska belica' (IB). The figure shows the seven most influential phenolic compounds, with coloured arrows from the most influential (red) to the least (blue).

The principal component analysis (PCA) of all analysed phenolic explains 52.1 % of the data variability with the two principal component (Fig. 4). Hydroxytyrosol, oleuropein aglycon, oleuropein, oleosid, hydroxytyrosol glucoside, elenolic acid and tyrosol were identified as the compounds responsible for the data distribution, which is mainly explained by cultivars. 'Istrska belica', with the longest processing period, differs the most from all cultivars, especially in the content of oleuropein and oleuropein aglycone. Cultivar 'Mata' also stands out from the other cultivars because of hydroxytyrosol, elenolic acid, tyrosol and hydroxytyrosol glucoside. The data from the PCA analysis also show that the cultivars with more grouped data had lower initial content of bitter compounds and therefore took less time to become non-bitter than others with more scattered data. However, in all cultivars, the harvest data strongly differ from the other data, which can suggest that major changes in phenolic composition occurred already in the first month of processing when the fruits were soaked in tap water. Therefore, it can be concluded that variations in phenolic compounds during the processing method are cultivar-specific, so that each cultivar would need to be considered individually for future method optimisations.

### 3.4 Sensory analysis

**Fig. 5:**
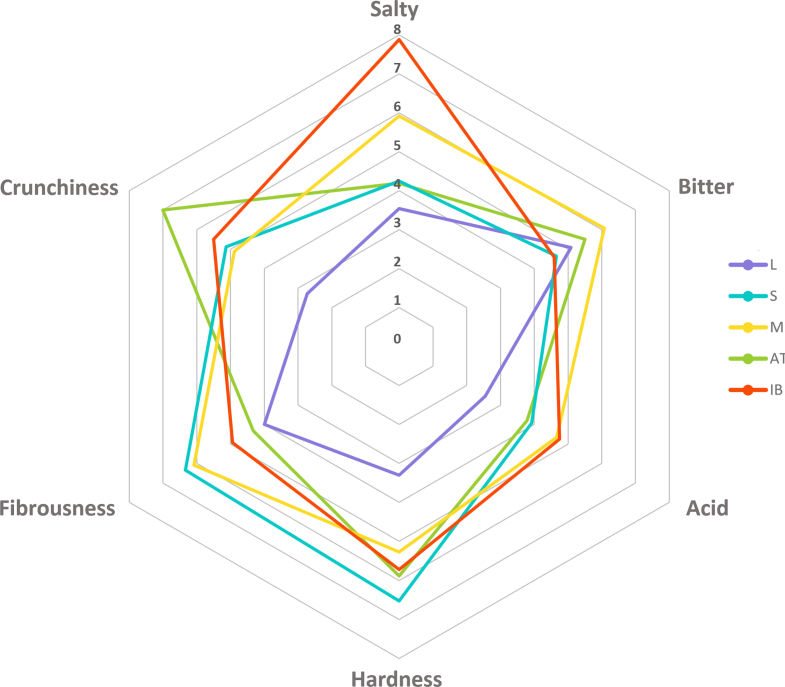
Sensory characteristic of cvs. 'Leccino' (L), 'Štorta' (S), 'Mata' (M), 'Ascolana tenera' (AT) and 'Istrska belica' (IB) at the end of processing.

Sensory studies of the final products did not show statistical difference in bitterness (Fig. 5, Table A.4), even the content of oleuropein and other bitter phenolic compound were different at the end of processing in different cultivars. 'Istrska belica' was found to be the saltiest cultivar, probably because it was exposed to brine for the longest time. According to the sensory analysis, 'Leccino' was the least acidic cultivar, which could be due to the shortest fermentation time, as the pH of the brine decreases with processing (Othman et al., 2009). The 'Ascolana tenera' was the crunchiest, in contrast to 'Leccino'. In terms of fruit size, the 'Ascolana tenera' had the largest fruits and 'Leccino' the smallest, which could affect the fruit crunchiness of the final product. Sensory analysis showed that 'Leccino' was the softest and 'Štorta' the hardest, which was also confirmed by firmness measurements of the final product. There were no statistical differences in fibrousness between the cultivars.

## 4 Conclusions

In this study, the variations in fruit quality parameters, antioxidant capacity and phenolic content during natural spontaneous fermentation of five Slovenian Istrian olive cultivars were analyzed in detail. The variations during the debittering process were monitored every month until the olives were ready for consumption. However, during processing the fruit weight and firmness decreased and dry matter increased in all cultivars, due to water movement from fruits to brine and degradation of fruits cell wall causing olives softening.

In this study, the variations in fourteen phenolic compounds were monitored. Nine of them (oleuropein aglycone, demethyoleuropein, elenolic acid, oleacein, oleoside, ligstroside, ligstroside agylicone, hydroxytyrosol glucoside and verbascoside) were studied for the first time during natural fermentation. The most abundant phenolic compound in olives was oleuropein, whose content decreased rapidly during processing in all cultivars. Its content at harvest was the highest in 'Istrska belica' and the lowest in 'Štorta', 'Leccino', so its content at the beginning of processing could be the main reason for its processing length. A decrease in oleuropein content could determine a decrease in antioxidant capacity, fruit browning and an increase in other phenolic compounds. Most of studied phenolic compounds (oleuropein aglycone, elenolic acid, oleoside, oleacein, hydroxytyrosol, hydroxytyrosol glucoside, verbascoside, caffeic acid and tyrosol) showed an increase in their content and when they reached their maximum they decreased, which was cultivar-specific. The contents of individual phenolic compounds during processing were cultivar specific. In general, the highest contents of phenolic compounds were observed in 'Istrska belica', 'Mata' and 'Ascolana tenera', which require the longest processing period and lower contents in 'Štorta' and 'Leccino', which require the shortest processing time to be ready for consumption. The content of the phenolic compounds, with bitter taste did not change significantly in the last sampling dates in all cultivars. Furthermore, sensory analysis of the final products showed that the bitter taste did not differ between cultivars, although the content of bitter phenolic compounds varied between cultivars. Understanding the detailed phenolic variations during the olive processing could reduce processing time, and lead to a higher content of phenolic compounds, which can extend shelf life, improve the nutritional value and quality of the final product. This optimization would improve efficiency and reduce water and salt consumption. However, our results show that variations in phenolic compounds during natural fermentation processing are cultivar-specific. Therefore, for a more accurate optimization, it would also be necessary to study the appropriate harvest time, the activity of enzymes, in particular *esterase* and *β-glucosidase*, the sensory evaluation during the last months of fermentation and the impact on consumer perception for each cultivar separately.

## Formatting of funding sources

This study is part of program P4-0013-0481, which is funded by Slovenian Research and Innovation Agency (ARIS).

## Ethical statement

We had confirmed that the appropriate protocols for protecting the rights and privacy of all participants were utilized in the execution of the sensory evaluation. The participants gave their consent to take part in the sensory study and use their information.

## CRediT authorship contribution statement

**Tea Burin:** Writing – original draft, Visualization, Resources, Methodology, Funding acquisition, Formal analysis, Data curation, Conceptualization. **Mariana Cecilia Grohar:** Methodology. **Jerneja Jakopic:** Methodology, Formal analysis. **Robert Veberic:** Visualization, Conceptualization. **Metka Hudina:** Visualization, Supervision, Conceptualization.

## Declaration of competing interest

The authors declare that they have no known competing financial interests or personal relationships that could have appeared to influence the work reported in this paper.

## Data Availability

Data will be made available on request.

## Appendix A Supplementary data

Supplementary material Image 1
