# Supplementary material for: Fermented olives (Olea europaea L.): A detailed insight into morphological changes and phenolic profile from harvest to jar
Source: Food Chem X. 2025 Feb 21;26:102309. doi: 10.1016/j.fochx.2025.102309 (PMC11910130; doi:10.1016/j.fochx.2025.102309)
Supplement: Supplementary file 1 — Supplementary material [file mmc1.docx]

| **Table A.1** Retention times (Rt), fragmentation data (MS^2^) of phenolic compounds in pseudo-molecular ion identified in negative ion mode [M−H]^−^. | | | | | |
| --- | --- | --- | --- | --- | --- |
| **Phenolic compound** | **Rt (min)** | **[M−H]^−^ (*m*/*z*)** | **MS² (*m*/*z*)** |  |  |
| Oleuropein | 23.5 | 539 | 377, 307, 275 |  |  |
| Oleuropein aglycone | 30.8 | 377 | 275, 307 |  |  |
| Hydroxytyrosol | 8.5 | 153 | 123, 109 |  |  |
| Oleoside-11-methyl ester | 13.6 | 403 | 223, 179, 195 |  |  |
| Elenolic acid | 8.6 | 241 | 197, 139 |  |  |
| Demethyloleuropein | 18.4 | 525 | 363, 919, 249 |  |  |
| Oleacein | 29.5 | 319 | 195, 183 |  |  |
| Oleoside | 11.3 | 389 | 345, 209, 371 |  |  |
| Ligstroside | 26.0 | 523 | 361, 112 |  |  |
| Ligstroside aglycone | 32.1 | 361 | 291, 259 |  |  |
| Hydroxytyrosol glucoside | 7.0 | 315 | 153 |  |  |
| Verbascoside | 21.3 | 623 | 461, 305, 443 |  |  |
| Caffeic acid | 15.3 | 179 | 135, 89, 99 |  |  |
| Tyrosol | 11.2 | 137 | 119 |  |  |

| **Table A.2** Fruit colour (L*, C*, h°) of the cultivars 'Leccino', 'Štorta', 'Ascolana tenera', 'Mata' and 'Istrska belica' during processing (from 0 to 12 months). | | | | | | |
| --- | --- | --- | --- | --- | --- | --- |
| Colour parameter | Processing time | Cultivar | | | | |
|  |  | 'Leccino' | 'Štorta' | 'Ascolana tenera' | 'Mata' | 'Istrska belica' |
| ****L*** | 0 | 20.20 ± 0.56 a | 47.97 ± 1.66 e | 54.33 ± 3.86 f | 56.45 ± 2.68 f | 49.71 ± 2.36 e |
|  | 1 | 20.69 ± 0.55 a | 45.09 ± 4.11 e | 42.01 ± 2.19 e | 48.23 ± 2.28 e | 42.85 ± 2.07 d |
|  | 2 | 23.97± 1.80 bc | 37.51 ± 2.36 bd | 37.23 ± 2.61 cd | 44.13 ± 3.75 d | 41.48 ± 2.94 cd |
|  | 3 | 24.40 ± 1.84 bc | 40.59 ± 2.79 d | 38.91 ± 2.72 d | 44.21 ± 3.50 d | 40.40 ± 4.08 bd |
|  | 4 | 25.05 ± 1.78 c | 38.25 ± 2.03 cd | 36.51 ± 2.19 bd | 42.23 ± 4.05 cd | 40.19 ± 3.12 bd |
|  | 5 | 23.34 ± 1.93 b | 35.37 ± 2.96 abc | 35.69 ± 1.80 bc | 42.52 ± 2.84 cd | 40.29 ± 2.98 bd |
|  | 6 | 23.36 ± 1.49 bc | 34.15 ± 3.77 ab | 35.67 ± 2.58 bc | 41.19 ± 4.5 cd | 37.19 ± 2.16 ab |
|  | 7 | 22.76 ± 1.24 b | 32.13 ± 3.35 a | 35.22 ± 2.34 bc | 38.92 ± 3.38 bc | 38.31 ± 2.10 abc |
|  | 8 |  |  | 35.15 ± 2.22 bc | 39.71 ± 3.03 bc | 37.45 ± 2.74 ab |
|  | 9 |  |  | 34.73 ± 3.14 bc | 39.15 ± 2.32 bc | 37.08 ± 2.95 ab |
|  | 10 |  |  | 34.23 ± 2.08 b | 36.05 ± 3.20 b | 36.70 ± 2.51 a |
|  | 11 |  |  | 31.01 ± 1.72 a | 31.88 ± 3.41 a | 35.93 ± 3.06 a |
|  | 12 |  |  |  |  | 35.92 ± 2.13 a |
| ****C*** | 0 | 3.23 ± 0.58 a | 37.83 ± 2.91 e | 37.43 ± 1.18 c | 39.02 ± 2.30 f | 37.11 ± 2.48 e |
|  | 1 | 4.33 ± 0.96 a | 24.02 ± 4.24 d | 23.39 ± 3.06 b | 29.45 ± 4.39 e | 27.17 ± 3.52 d |
|  | 2 | 6.96 ± 1.51 b | 21.59 ± 4.42 bd | 12.15 ± 2.42 a | 28.33 ± 4.92 de | 24.45 ± 4.51 cd |
|  | 3 | 6.92 ± 1.14 b | 22.77 ± 5.09 cd | 13.27 ± 2.52 a | 23.70 ± 4.65 cd | 23.51 ± 4.36 bd |
|  | 4 | 7.39 ± 1.59 bc | 18.57 ± 2.67 ab | 12.13 ± 2.40 a | 22.17 ± 5.32 bc | 23.29 ± 4.34 bd |
|  | 5 | 7.54 ± 2.39 bc | 18.83 ± 3.92 abc | 14.73 ± 3.16 a | 20.93 ± 5.85 bc | 23.69 ± 3.83 bd |
|  | 6 | 9.23 ± 2.43 cd | 14.95 ± 2.88 a | 13.13 ± 1.93 a | 21.19 ± 5.26 bc | 21.54 ± 5.05 abc |
|  | 7 | 9.72 ± 2.86 d | 15.71 ± 2.5 a | 13.16 ± 2.36 a | 19.60 ± 4.19 bc | 21.21 ± 2.52 abc |
|  | 8 |  |  | 14.49 ± 2.10 a | 18.11 ± 3.48 ab | 20.79 ± 3.43 abc |
|  | 9 |  |  | 12.47 ± 2.90 a | 18.94 ± 2.77 bc | 19.59 ± 1.84 abc |
|  | 10 |  |  | 13.29 ± 2.49 a | 18.59 ± 4.50 bc | 18.55 ± 3.63 a |
|  | 11 |  |  | 14.61 ± 2.72 a | 13.28 ± 2.47 a | 17.85 ± 4.05 a |
|  | 12 |  |  |  |  | 18.15 ± 3.39 a |
| ***h°*** | 0 | 49.85 ± 5.60 d | 94.14 ± 1.86 e | 97.09 ± 2.24 d | 94.51 ± 1.51 g | 98.73 ± 0.91 f |
|  | 1 | 45.90 ± 5.74 cd | 71.69 ± 4.15 d | 73.78 ± 2.44 c | 77.75 ± 1.82 f | 76.22 ± 2.44 e |
|  | 2 | 45.68 ± 2.82 cd | 68.11 ± 4.85 cd | 63.27 ± 3.04 b | 71.42 ± 3.80 e | 70.14 ± 2.49 cd |
|  | 3 | 45.40 ± 2.84 cd | 68.75 ± 3.77 cd | 61.30 ± 3.94 ab | 69.45 ± 2.46 de | 70.34 ± 2.78 d |
|  | 4 | 44.26 ± 7.54 bcd | 65.10 ± 5.72 ac | 60.14 ± 3.48 ab | 67.98 ± 4.57 cde | 70.26 ± 2.58 cd |
|  | 5 | 40.42 ± 4.50 ac | 65.67 ± 3.71 bc | 61.74 ± 3.40 ab | 68.09 ± 5.4 cde | 70.27 ± 2.62 cd |
|  | 6 | 39.48 ± 6.53 ab | 60.21 ± 4.94 a | 60.57 ± 3.97 ab | 67.66 ± 5.22 be | 70.16 ± 2.47 cd |
|  | 7 | 37.29 ± 3.19 b | 61.99 ± 5.99 abc | 61.13 ± 5.72 ab | 65.21 ± 5.69 bd | 69.45 ± 2.04 bd |
|  | 8 |  |  | 60.33 ± 5.72 ab | 63.67 ± 4.21 bc | 68.07 ± 3.22 bd |
|  | 9 |  |  | 61.44 ± 4.55 ab | 65.77 ± 2.49 bd | 66.40 ± 2.54 ab |
|  | 10 |  |  | 61.36 ± 3.25 ab | 62.75 ± 5.35 ab | 66.95 ± 3.19 bc |
|  | 11 |  |  | 58.36 ± 4.59 a | 58.23 ± 4.80 a | 66.31 ± 1.69 ab |
|  | 12 |  |  |  |  | 63.21 ± 4.95 a |
| The values are means ± SD. Different letters indicate statistical differences among different month of processing according to Tukey’s multiple range test (*p* ≤ 0.05). | | | | | | |

| **Table A.3** Individual phenolic compounds during processinf (from 0 to 12 months) of the cultivars 'Leccino', 'Štorta', 'Ascolana tenera', 'Mata' and 'Istrska belica'. | | | | | | |
| --- | --- | --- | --- | --- | --- | --- |
| Phenolic compound | Processing time | Cultivar | | | | |
|  |  | **'Leccino'** | **'Štorta'** | **'Ascolana tenera'** | **'Mata'** | **'Istrska belica'** |
| **Oleuropein** | 0 | 9802.19 ± 96.28 Aa | 7633.57 ± 458.12 Aa | 11877.21 ± 1364.97 aA | 34587.54 ± 1656.82 aC | 57694.04 ± 3642.23 aB |
|  | 1 | 1167.99 ± 110.02 b | 2406.17 ± 453.38 b | 3307.56 ± 98.31 d | 9801.76 ± 262.87 b | 28432.06 ± 1712.31 b |
|  | 2 | 620.82 ± 52.94 c | 765.70 ± 72.18 c | 2400.56 ± 106.00 dc | 6741.89 ± 631.75 c | 24532.29 ± 1916.71 b |
|  | 3 | 321.12 ± 40.01 d | 385.82 ± 19.38 cd | 2119.47 ± 161.69 c | 5099.97 ± 418.15 cd | 19535.49 ± 2873.34 c |
|  | 4 | 194.23 ± 38.56 de | 303.45 ± 21.33 cd | 1529.44 ± 64.22 cd | 5431.88 ± 467.43 cd | 19325.35 ± 1248.09 c |
|  | 5 | 185.67 ± 30.89 de | 309.05 ± 23.71 cd | 1459.71 ± 25.05 cd | 3831.49 ± 712.68 de | 15213.32 ± 236.87 cd |
|  | 6 | 170.59 ± 17.36 de | 239.81 ± 7.31 cd | 926.47 ± 107.38 d | 1382.47 ± 118.24 e | 13304.10 ± 800.44 de |
|  | 7 | 32.63 ± 2.14 e, A | 98.41 ± 31.83 d, A | 576.25 ± 51.52 d | 1296.92 ± 54.96 e | 10255.66 ± 990.36 ef |
|  | 8 |  |  | 483.96 ± 80.93 d | 1028.61 ± 31.83 e | 8246.06 ± 846.05 fg |
|  | 9 |  |  | 482.20 ± 90.61 d | 935.05 ± 72.50 e | 7613.22 ± 712.52 fh |
|  | 10 |  |  | 509.06 ± 85.63 d | 833.02 ± 84.62 e | 5235.91 ± 1154.23 ghi |
|  | 11 |  |  | 375.57 ± 33.27 d, A | 191.23 ± 47.19 e, A | 3006.55 ± 283.87 hi |
|  | 12 |  |  |  |  | 1520.2 ± 379.79 i, B |
| **Oleuropein aglicone** | 0 | 184.69 ± 34.86 a, C | 31.84 ± 3.07 a, A | 66.02 ± 10.66 ab, A | 662.88 ± 7.65 a, D | 1287.23 ± 57.96 a, B |
|  | 1 | 366.76 ± 20.57 b | 1998.64 ± 140.42 b | 84.05 ± 12.62 a | 1569.89 ± 111.97 b | 2576.75 ± 117.54 bc |
|  | 2 | 54.18 ± 3.71 cd | 3429.81 ± 75.59 c | 221.92 ± 31.49 c | 832.77 ± 38.61 c | 2849.95 ± 114.59 bd |
|  | 3 | 61.96 ± 1.40 c | 295.60 ± 35.76 d | 35.09 ± 11.00 bd | 696.51 ± 27.43 a | 3450.45 ± 125.87 e |
|  | 4 | 24.57 ± 6.37 cd | 153.07 ± 8.77 ad | 25.36 ± 3.90 d | 499.03 ± 52.36 d | 4405.06 ± 143.95 f |
|  | 5 | 20.28 ± 0.55 d | 561.23 ± 25.50 e | 23.55 ± 1.27 d | 434.30 ± 6.32 de | 3388.86 ± 163.46 eg |
|  | 6 | 19.40 ± 1.01 d | 80.87 ± 5.38 a | 20.08 ± 2.71 d | 325.73 ± 10.19 ef | 3129.94 ± 109.69 dg |
|  | 7 | 13.24 ± 0.98 d, A | 29.99 ± 1.00 a, A | 20.48 ± 2.72 d | 314.28 ± 26.05 ef | 2552.33 ± 111.34 c |
|  | 8 |  |  | 20.18 ± 4.66 d | 170.19 ± 15.52 g | 1089.94 ± 31.10 a |
|  | 9 |  |  | 12.86 ± 2.08 d | 152.73 ± 6.30 g | 1087.78 ± 44.15 a |
|  | 10 |  |  | 11.41 ± 1.68 d | 221.93 ± 32.78 fg | 1137.66 ± 54.93 a |
|  | 11 |  |  | 8.40 ± 1.91 d, A | 335.09 ± 49.85 ef, C | 515.87 ± 32.72 h |
|  | 12 |  |  |  |  | 341.39 ± 62.79 h, B |
| **Hydroxytyrosol** | 0 | 303.36 ± 24.83 a, C | 7.24 ± 0.90 a, A | 30.38 ± 7.49 a, A | 179.32 ± 1.03 a, D | 87.33 ± 10.97 a, B |
|  | 1 | 362.89 ± 11.15 b | 169.56 ± 9.60 b | 335.15 ± 8.43 b | 684.05 ± 27.96 b | 640.08 ± 22.19 bc |
|  | 2 | 349.04 ± 10.99 b | 116.15 ± 5.11 c | 648.20 ± 10.49 c | 1172.97 ± 37.40 c | 665.90 ± 48.29 bc |
|  | 3 | 339.01 ± 13.53 ab | 34.16 ± 4.03 a | 654.05 ± 28.78 c | 1173.51 ± 40.44 c | 730.95 ± 27.81 bd |
|  | 4 | 259.21 ± 19.31 c | 16.27 ± 1.25 a | 979.40 ± 33.82 d | 1225.66 ± 61.99 cd | 763.91 ± 9.16 d |
|  | 5 | 227.61 ± 14.20 c | 711.91 ± 5.69 d | 961.53 ± 10.23 d | 1203.11 ± 76.46 b | 937.01 ± 42.99 e |
|  | 6 | 68.62 ± 4.41 d | 6.65 ± 0.21 a | 753.49 ± 29.01 e | 636.90 ± 45.55 d | 706.93 ± 26.82 cd |
|  | 7 | 15.47 ± 0.85 e, C | 6.83 ± 0.54 a, C | 686.79 ± 53.45 ce | 1297.58 ± 11.89 b | 869.56 ± 46.39 e |
|  | 8 |  |  | 564.43 ± 14.76 f | 657.91 ± 41.53 e | 620.69 ± 32.30 c |
|  | 9 |  |  | 520.22 ± 29.06 fg | 503.38 ± 11.51 e | 495.86 ± 17.26 f |
|  | 10 |  |  | 465.42 ± 47.92 g | 451.95 ± 23.17 e | 447.91 ± 12.61 fg |
|  | 11 |  |  | 296.71 ± 3.56 b, A | 429.33 ± 4.77 e, D | 402.14 ± 49.31 gh |
|  | 12 |  |  |  |  | 353.85 ± 21.04 h, B |
| **Oleoside-11-methyl ester** | 0 | 255.56 ± 10.07 c, A | 32.20 ± 0.31 a, D | 182.43 ± 8.84 a, A | 115.38 ± 8.64 ab, B | 129.17 ± 8.79 a, B |
|  | 1 | 286.48 ± 23.00 ab | 30.99 ± 1.19 a | 207.31 ± 9.78 ab | 134.87 ± 11.62 ac | 134.29 ± 6.15 ab |
|  | 2 | 282.92 ± 19.83 ab | 62.91 ± 3.11 b | 210.08 ± 14.28 b | 133.43 ± 1.85 ac | 139.82 ± 7.47 abc |
|  | 3 | 293.34 ± 12.94 b | 5.17 ± 0.36 c | 223.62 ± 10.44 bc | 144.55 ± 6.10 c | 136.01 ± 7.05 ab |
|  | 4 | 277.45 ± 9.54 ab | 6.20 ± 1.30 c | 231.44 ± 4.42 bd | 155.80 ± 12.20 c | 150.03 ± 5.46 abd |
|  | 5 | 290.30 ± 4.27 ab | 53.61 ± 4.96 d | 239.59 ± 4.82 cd | 184.99 ± 15.09 d | 157.54 ± 7.19 be |
|  | 6 | 293.56 ± 8.64 b | 3.21 ± 0.61 c | 227.25 ± 4.25 bc | 101.39 ± 4.94 be | 155.40 ± 1.83 be |
|  | 7 | 332.55 ± 1.36 c, C | 3.22 ± 0.68 c, E | 241.84 ± 9.65 cdef | 98.48 ± 4.39 be | 163.20 ± 7.58 cdef |
|  | 8 |  |  | 244.40 ± 4.75 cd | 80.27 ± 8.40 ef | 165.58 ± 10.05 def |
|  | 9 |  |  | 249.01 ± 4.25 d | 71.00 ± 3.02 f | 174.70 ± 7.48 ef |
|  | 10 |  |  | 238.39 ± 9.02 cd | 109.74 ± 11.22 ab | 177.96 ± 12.89 ef |
|  | 11 |  |  | 242.2 ± 10.00 cd, A | 117.67 ± 1.11 ab, D | 186.72 ± 5.20 f |
|  | 12 |  |  |  |  | 184.17 ± 12.65 f, B |
| **Elenolic acid** | 0 | 29.23 ± 8.42 a, AC | 0.00 ± 0.00 a, B | 19.41 ± 4.12 a, A | 35.37 ± 2.18 a, C | 0.00 ± 0.00 a, B |
|  | 1 | 51.99 ± 7.84 b | 0.00 ± 0.00 a | 85.88 ± 2.87 bc | 42.80 ± 2.92 a | 0.00 ± 0.00 a |
|  | 2 | 53.61 ± 7.65 b | 0.00 ± 0.00 a | 149.90 ± 8.90 de | 166.99 ± 17.11 bc | 0.00 ± 0.00 a |
|  | 3 | 82.18 ± 4.30 c | 0.00 ± 0.00 a | 139.10 ± 11.31 df | 264.05 ± 14.66 de | 63.73 ± 11.02 bc |
|  | 4 | 116.21 ± 7.38 d | 0.00 ± 0.00 a | 170.55 ± 17.87 dfg | 264.28 ± 25.28 de | 120.86 ± 7.74 d |
|  | 5 | 108.92 ± 4.00 de | 0.00 ± 0.00 a | 207.40 ± 7.75 gh | 308.00 ± 28.50 df | 188.19 ± 20.73 e |
|  | 6 | 91.38 ± 5.31 ce | 11.23 ± 1.02 b | 280.98 ± 38.83 i | 316.41 ± 34.91 f | 215.52 ± 14.91 e |
|  | 7 | 93.61 ± 6.48 ce, C | 11.87 ± 0.88 b, B | 327.04 ± 12.87 j | 386.29 ± 13.26 g | 144.43 ± 4.96 d |
|  | 8 |  |  | 235.95 ± 15.60 h | 216.18 ± 6.51 be | 129.36 ± 9.81 d |
|  | 9 |  |  | 193.54 ± 9.42 dh | 165.06 ± 6.59 bc | 76.82 ± 12.88 bc |
|  | 10 |  |  | 122.61 ± 10.12 be | 161.92 ± 13.14 c | 45.72 ± 5.21 c |
|  | 11 |  |  | 74.95 ± 5.25 c, A | 63.78 ± 10.38 a, A | 0.00 ± 0.00 a |
|  | 12 |  |  |  |  | 0.00 ± 0.00 a, B |
| **Demethyloleuropein** | 0 | 180.26 ± 9.60 a, C | 268.86 ± 4.72 a, D | 21.03 ± 0.42 a, A | 5.20 ± 0.64 a, B | 15.37 ± 0.50 a, AB |
|  | 1 | 88.36 ± 10.52 b | 65.51 ± 8.79 b | 23.71 ± 1.15 a | 9.94 ± 1.02 bc | 2.80 ± 2.59 b |
|  | 2 | 36.35 ± 0.16 c | 22.27 ± 1.15 c | 31.71 ± 1.01 bc | 20.08 ± 0.64 d | 94.25 ± 11.90 c |
|  | 3 | 46.65 ± 10.71 c | 13.67 ± 0.76 cd | 32.09 ± 1.66 bc | 20.14 ± 1.18 d | 119.44 ± 1.98 d |
|  | 4 | 46.88 ± 7.26 c | 12.35 ± 1.03 cd | 35.41 ± 3.36 b | 23.24 ± 1.50 d | 136.27 ± 6.73 de |
|  | 5 | 35.11 ± 5.30 c | 11.98 ± 0.86 d | 29.77 ± 0.40 cd | 33.75 ± 3.28 e | 157.65 ± 7.49 f |
|  | 6 | 3.94 ± 0.49 d | 3.56 ± 0.23 de | 35.63 ± 0.41 b | 14.01 ± 0.58 f | 145.46 ± 11.80 ef |
|  | 7 | 1.68 ± 0.18 d, B | 0.00 ± 0.00 e, B | 32.92 ± 1.80 bd | 13.51 ± 0.48 cf | 137.55 ± 11.80 df |
|  | 8 |  |  | 29.69 ± 1.27 cd | 11.78 ± 0.36 cf | 134.30 ± 2.36 de |
|  | 9 |  |  | 28.52 ± 0.56 c | 6.99 ± 0.31 ab | 65.04 ± 3.00 bc |
|  | 10 |  |  | 30.02 ± 0.89 cd | 5.65 ± 1.05 a | 50.61 ± 3.89 bg |
|  | 11 |  |  | 22.02 ± 1.07 a, A | 3.42 ± 0.45 a, B | 37.38 ± 2.90 gh |
|  | 12 |  |  |  |  | 23.54 ± 4.87 ah, A |
| **Oleac**  **ein** | 0 | 3.79 ± 0.43 a, B | 0.00 ± 0.00 a, A | 0.00 ± 0.00 a, A | 4.55 ± 0.00 a, B | 0.00 ± 0.00 a, A |
|  | 1 | 12.22 ± 0.96 a | 28.25 ± 3.67 ab | 37.10 ± 0.34 b | 3.91 ± 8.91 b | 153.86 ± 3.93 b |
|  | 2 | 47.89 ± 5.73 b | 51.34 ± 9.69 b | 84.44 ± 8.30 c | 3.71 ± 9.55 c | 148.78 ± 6.50 b |
|  | 3 | 59.72 ± 3.94 cd | 49.94 ± 1.19 b | 102.60 ± 5.25 cd | 3.75 ± 5.82 cd | 178.92 ± 6.20 b |
|  | 4 | 51.36 ± 5.01 bc | 50.83 ± 3.93 b | 106.96 ± 8.58 cd | 4.55 ± 20.75 cd | 204.99 ± 8.75 bc |
|  | 5 | 60.93 ± 1.02 d | 398.29 ± 25.65 c | 120.21 ± 7.59 def | 4.53 ± 20.66 def | 212.23 ± 13.74 bc |
|  | 6 | 65.93 ± 1.01 d | 86.74 ± 12.29 d | 126.67 ± 9.55 df | 4.39 ± 36.84 ef | 234.5 ± 31.62 bc |
|  | 7 | 122.16 ± 3.52 e, A | 27.39 ± 0.59 ab, D | 137.55 ± 6.64 f | 4.3 ± 24.34 g | 473.07 ± 26.25 d |
|  | 8 |  |  | 126.23 ± 5.77 df | 4.12 ± 24.08 h | 763.19 ± 36.26 e |
|  | 9 |  |  | 143.01 ± 2.19 f | 4.12 ± 46.45 f | 948.68 ± 63.94 f |
|  | 10 |  |  | 184.56 ± 18.79 g | 3.89 ± 3.66 d | 774.78 ± 71.86 e |
|  | 11 |  |  | 100.66 ± 8.97 ce, A | 4.17 ± 16.31 de, C | 485.05 ± 35.64 de |
|  | 12 |  |  |  |  | 304.64 ± 33.18 c, B |
| **Oleoside** | 0 | 8.00 ± 1.16 a, C | 31.76 ± 1.18 a, A | 26.37 ± 2.40 a, A | 190.16 ± 14.05 a, D | 57.10 ± 2.54 a, B |
|  | 1 | 179.67 ± 19.87 b | 65.70 ± 3.66 b | 51.77 ± 4.50 ab | 412.29 ± 19.55 b | 218.90 ± 22.45 b |
|  | 2 | 315.65 ± 18.88 c | 97.51 ± 5.37 c | 72.13 ± 4.85 bc | 413.56 ± 20.98 b | 377.89 ± 10.86 c |
|  | 3 | 21.61 ± 1.32 a | 22.41 ± 1.70 de | 94.31 ± 4.27 cd | 339.19 ± 13.18 cd | 475.61 ± 11.97 de |
|  | 4 | 17.76 ± 2.51 a | 11.01 ± 0.54 f | 108.01 ± 4.01 d | 238.07 ± 10.42 de | 658.06 ± 37.55 e |
|  | 5 | 14.71 ± 1.63 a | 41.95 ± 2.97 g | 208.12 ± 5.10 e | 242.31 ± 19.69 df | 532.15 ± 23.30 f |
|  | 6 | 13.18 ± 0.61 a | 24.02 ± 0.90 ad | 303.98 ± 13.46 f | 172.34 ± 1.87 a | 426.91 ± 13.38 cd |
|  | 7 | 10.88 ± 1.33 a, B | 15.72 ± 2.37 ef, B | 245.35 ± 17.88 g | 399.20 ± 26.29 b | 413.24 ± 11.41 c |
|  | 8 |  |  | 219.90 ± 11.56 eg | 250.82 ± 6.42 d | 383.52 ± 3.69 c |
|  | 9 |  |  | 224.69 ± 3.07 eg | 202.36 ± 2.38 aef | 272.71 ± 3.61 g |
|  | 10 |  |  | 148.22 ± 21.87 h | 198.61 ± 3.60 ae | 247.12 ± 17.06 bg |
|  | 11 |  |  | 115.02 ± 3.95 d, A | 176.16 ± 3.83 a, C | 151.84 ± 16.22 h |
|  | 12 |  |  |  |  | 135.19 ± 13.44 h, A |
| **Ligstroside aglycone** | 0 | 33.71 ± 3.87 a, A | 87.67 ± 1.45 a, C | 24.41 ± 2.20 ab, A | 241.88 ± 12.43 a, D | 67.2 ± 5.14 a, B |
|  | 1 | 5.68 ± 0.38 b | 99.84 ± 6.36 a | 35.32 ± 3.42 ac | 198.72 ± 9.68 b | 66.34 ± 8.44 ab |
|  | 2 | 6.19 ± 0.21 b | 107.76 ± 10.45 a | 40.25 ± 6.02 c | 185.46 ± 4.65 bc | 95.16 ± 2.79 c |
|  | 3 | 7.77 ± 0.99 b | 94.63 ± 6.34 a | 40.41 ± 3.89 c | 163.04 ± 10.03 c | 64.53 ± 5.32 ad |
|  | 4 | 29.03 ± 2.48 a | 85.43 ± 9.95 a | 40.14 ± 5.69 c | 113.81 ± 8.24 d | 50.30 ± 5.34 def |
|  | 5 | 23.54 ± 0.43 c | 95.79 ± 14.06 a | 39.03 ± 7.57 ac | 104.54 ± 19.83 d | 59.84 ± 7.00 af |
|  | 6 | 18.28 ± 1.21 d | 97.39 ± 14.64 a | 40.11 ± 6.09 c | 88.95 ± 11.12 d | 57.33 ± 6.26 af |
|  | 7 | 17.58 ± 0.69 d, A | 96.03 ± 4.27 a, C | 27.87 ± 8.46 bc | 48.43 ± 24.24 e | 50.98 ± 4.64 bdef |
|  | 8 |  |  | 19.98 ± 1.48 b | 39.33 ± 6.07 e | 48.58 ± 1.36 fg |
|  | 9 |  |  | 24.34 ± 4.02 ab | 36.17 ± 6.27 e | 51.73 ± 6.66 aef |
|  | 10 |  |  | 19.91 ± 5.05 b | 38.53 ± 0.67 e | 45.75 ± 1.01 fg |
|  | 11 |  |  | 19.61 ± 3.17 b, A | 33.70 ± 4.69 e, B | 37.96 ± 5.43 eg |
|  | 12 |  |  |  |  | 34.36 ± 2.52 g, B |
| **Ligstroside** | 0 | 33.71 ± 3.87 a, A | 127.76 ± 8.54 a, D | 26.35 ± 1.13 a, A | 883.84 ± 8.81 a, C | 726.32 ± 47.54 a, B |
|  | 1 | 3.13 ± 0.36 b | 0.00 ± 0.00 b | 0.00 ± 0.00 b | 109.95 ± 0.72 b | 330.36 ± 19.79 b |
|  | 2 | 0.00 ± 0.00 b | 0.00 ± 0.00 b | 0.00 ± 0.00 b | 53.40 ± 3.61 c | 264.91 ± 11.45 c |
|  | 3 | 0.00 ± 0.00 b | 0.00 ± 0.00 b | 0.00 ± 0.00 b | 40.19 ± 0.92 cd | 225.88 ± 21.00 cd |
|  | 4 | 0.00 ± 0.00 b | 0.00 ± 0.00 b | 0.00 ± 0.00 b | 39.74 ± 7.65 cd | 195.23 ± 11.49 d |
|  | 5 | 0.00 ± 0.00 b | 0.00 ± 0.00 b | 0.00 ± 0.00 b | 25.69 ± 5.98 de | 192.13 ± 13.94 de |
|  | 6 | 0.00 ± 0.00 b | 0.00 ± 0.00 b | 0.00 ± 0.00 b | 12.87 ± 0.34 de | 124.31 ± 5.96 e |
|  | 7 | 0.00 ± 0.00 b, A | 0.00 ± 0.00 b, A | 0.00 ± 0.00 b | 0.00 ± 0.00 e | 107.57 ± 3.76 e |
|  | 8 |  |  | 0.00 ± 0.00 b | 0.00 ± 0.00 e | 81.29 ± 19.54 ef |
|  | 9 |  |  | 0.00 ± 0.00 b | 0.00 ± 0.00 e | 47.01 ± 4.37 fg |
|  | 10 |  |  | 0.00 ± 0.00 b | 0.00 ± 0.00 e | 37.09 ± 3.61 fg |
|  | 11 |  |  | 0.00 ± 0.00 b, A | 0.00 ± 0.46 e, A | 24.84 ± 2.91 g |
|  | 12 |  |  |  |  | 14.74 ± 1.33 g, B |
| **Hydroxyyrosol glucoside** | 0 | 105.80 ± 5.19 a, C | 12.20 ± 0.50 a, B | 23.71 ± 3.29 a, A | 45.82 ± 5.23 a, D | 14.21 ± 1.55 a, AB |
|  | 1 | 41.08 ± 5.00 b | 34.63 ± 2.36 b | 87.93 ± 6.67 b | 311.41 ± 18.98 b | 94.73 ± 5.41 b |
|  | 2 | 17.93 ± 3.06 cd | 36.41 ± 2.56 b | 35.84 ± 3.81 a | 312.48 ± 12.05 b | 97.40 ± 9.60 bc |
|  | 3 | 22.29 ± 1.34 c | 38.64 ± 1.27 b | 37.80 ± 6.10 a | 295.55 ± 21.57 b | 97.57 ± 1.16 bc |
|  | 4 | 7.81 ± 0.72 e | 38.44 ± 1.72 b | 62.27 ± 2.88 c | 279.03 ± 14.64 b | 110.64 ± 2.09 cd |
|  | 5 | 11.56 ± 1.01 de | 61.89 ± 2.14 c | 69.07 ± 3.16 cd | 233.20 ± 28.29 c | 114.10 ± 9.85 d |
|  | 6 | 5.79 ± 0.66 e | 2.85 ± 0.18 d | 111.18 ± 7.42 e | 133.52 ± 15.26 d | 94.31 ± 7.53 b |
|  | 7 | 6.97 ± 0.21 e, C | 0.83 ± 0.33 d, C | 83.75 ± 7.29 bd | 109.99 ± 1.65 de | 93.75 ± 3.58 b |
|  | 8 |  |  | 70.26 ± 4.89 cd | 86.01 ± 5.51 ae | 67.57 ± 0.32 e |
|  | 9 |  |  | 67.21 ± 5.91 c | 77.56 ± 2.69 ae | 50.21 ± 1.88 f |
|  | 10 |  |  | 57.68 ± 4.44 c | 79.56 ± 12.58 ae | 46.72 ± 3.85 f |
|  | 11 |  |  | 38.23 ± 4.33 a, A | 64.03 ± 5.51 a, D | 30.37 ± 3.52 g |
|  | 12 |  |  |  |  | 23.66 ± 2.80 ag, B |
| **Verbascoside** | 0 | 618.79 ± 16.55 a, C | 12.37 ± 2.24 a, D | 938.69 ± 32.32 a, A | 104.32 ± 5.25 a, B | 77.85 ± 2.99 a, B |
|  | 1 | 617.97 ± 70.06 a | 211.25 ± 13.68 b | 858.36 ± 17.42 b | 274.38 ± 15.33 b | 502.26 ± 39.13 b |
|  | 2 | 535.01 ± 37.31 ab | 92.00 ± 3.91 c | 859.15 ± 9.82 b | 639.48 ± 4.71 c | 577.18 ± 51.15 bc |
|  | 3 | 528.34 ± 30.77 b | 57.69 ± 1.30 d | 751.02 ± 23.07 c | 629.82 ± 41.32 c | 817.24 ± 28.79 de |
|  | 4 | 480.28 ± 6.36 b | 63.02 ± 2.31 d | 718.37 ± 20.97 cd | 627.71 ± 29.98 c | 940.23 ± 24.49 d |
|  | 5 | 348.09 ± 10.67 c | 169.70 ± 4.10 e | 670.68 ± 18.18 de | 315.38 ± 33.28 b | 940.80 ± 67.16 d |
|  | 6 | 326.02 ± 11.43 c | 30.97 ± 5.55 f | 638.82 ± 34.90 e | 306.23 ± 31.95 b | 724.48 ± 29.84 ef |
|  | 7 | 298.09 ± 9.27 c, C | 9.32 ± 0.80 a, A | 543.56 ± 59.19 f | 618.45 ± 27.60 c | 687.18 ± 34.20 cef |
|  | 8 |  |  | 482.14 ± 1.56 fg | 438.92 ± 27.70 d | 764.73 ± 62.69 e |
|  | 9 |  |  | 412.26 ± 4.83 gh | 445.22 ± 17.16 d | 739.77 ± 56.84 ef |
|  | 10 |  |  | 358.78 ± 28.27 h | 428.50 ± 27.63 d | 617.10 ± 50.60 bf |
|  | 11 |  |  | 60.09 ± 21.24 i, A | 471.82 ± 25.31 d, B | 552.15 ± 51.08 bc |
|  | 12 |  |  |  |  | 492.56 ± 70.63 b, B |
| **Caffeic acid** | 0 | 10.25 ± 0.77 a, A | 1.51 ± 3.29 a, D | 7.91 ± 1.32 a, A | 47.67 ± 0.78 ab, C | 17.34 ± 4.11 a, B |
|  | 1 | 21.49 ± 1.19 b | 30.68 ± 6.67 b | 20.70 ± 1.07 b | 91.95 ± 7.00 c | 5.01 ± 1.47 ab |
|  | 2 | 23.18 ± 2.60 b | 21.99 ± 3.80 c | 22.32 ± 2.89 b | 83.48 ± 5.81 d | 2.48 ± 0.93 ac |
|  | 3 | 26.78 ± 5.16 bc | 23.15 ± 6.09 c | 29.26 ± 2.37 c | 63.21 ± 1.81 b | 1.28 ± 2.18 a |
|  | 4 | 29.06 ± 1.21 bc | 22.14 ± 2.88 c | 32.38 ± 2.14 cd | 57.05 ± 6.35 be | 9.54 ± 1.53 bcd |
|  | 5 | 32.01 ± 2.83 c | 45.09 ± 3.15 d | 33.84 ± 0.40 ce | 60.16 ± 3.02 be | 3.12 ± 3.21 d |
|  | 6 | 33.31 ± 3.72 c | 13.65 ± 7.41 e | 31.79 ± 1.60 c | 41.51 ± 16.30 ae | 0.69 ± 1.00 bd |
|  | 7 | 42.06 ± 0.68 d, A | 4.82 ± 7.29 a, C | 32.03 ± 2.03 c | 4.06 ± 8.13 b | 7.49 ± 2.69 de |
|  | 8 |  |  | 37.22 ± 1.35 def | 9.46 ± 2.49 ab | 1.21 ± 2.96 def |
|  | 9 |  |  | 37.87 ± 1.15 ef | 1.07 ± 3.45 be | 4.69 ± 3.25 f |
|  | 10 |  |  | 40.61 ± 1.06 f | 1.44 ± 6.90 ab | 4.31 ± 2.24 f |
|  | 11 |  |  | 40.45 ± 0.36 f, A | 4.12 ± 0.76 a, C | 8.68 ± 3.96 f |
|  | 12 |  |  |  |  | 5.67 ± 2.89 g, B |
| **Tyrosol** | 0 | 223.04 ± 12.55 a, C | 120.68 ± 3.52 a, E | 20.46 ± 2.41 a, A | 93.91 ± 4.28 a, D | 58.15 ± 1.58 ab, B |
|  | 1 | 155.25 ± 10 b | 62.58 ± 1.37 b | 65.01 ± 1.66 bc | 152.45 ± 13.64 b | 116.97 ± 9.66 cd |
|  | 2 | 134.38 ± 9.19 bc | 56.18 ± 3.06 b | 100.73 ± 2.75 de | 191.20 ± 4.00 c | 88.22 ± 20.08 bde |
|  | 3 | 115.70 ± 5.01 c | 36.96 ± 6.64 c | 119.31 ± 5.44 d | 142.81 ± 5.48 b | 83.16 ± 41.63 bde |
|  | 4 | 67.56 ± 6.74 df | 32.83 ± 3.13 c | 114.76 ± 11.23 df | 148.39 ± 7.34 b | 140.16 ± 20.75 cf |
|  | 5 | 50.23 ± 4.28 de | 82.83 ± 5.93 d | 111.94 ± 8.09 df | 138.33 ± 11.39 b | 126.51 ± 11.18 ce |
|  | 6 | 39.36 ± 2.75 ef | 18.33 ± 2.59 e | 91.21 ± 8.55 bde | 108.56 ± 8.79 a | 121.73 ± 28.23 cd |
|  | 7 | 27.39 ± 1.99 f, C | 10.83 ± 0.70 e, D | 85.20 ± 5.63 bef | 208.91 ± 11.13 c | 144.34 ± 11.73 c |
|  | 8 |  |  | 63.82 ± 9.73 bc | 137.92 ± 12.30 b | 105.34 ± 5.90 acd |
|  | 9 |  |  | 75.83 ± 0.21 ce | 107.81 ± 3.93 a | 91.51 ± 2.35 bdef |
|  | 10 |  |  | 52.70 ± 4.54 ac | 99.18 ± 11.73 a | 79.93 ± 4.13 bde |
|  | 11 |  |  | 68.61 ± 8.3 ce, A | 64.51 ± 3.77 d, AB | 71.26 ± 3.74 bd |
|  | 12 |  |  |  |  | 52.95 ± 6.22 b, B |
| The values are means ± SD. Different small letters indicate statistical differences among different month of processing and capital letters indicate statistical differences among different cultivars at harvest and final product, according to Tukey’s multiple range tes (*p* ≤ 0.05). | | | | | | |

| **Table A.4** Sensory analysis of the final product of the cultivars 'Leccino', 'Štorta', 'Ascolana tenera', 'Mata' and 'Istrska belica'. | | | | |  |  |  |
| --- | --- | --- | --- | --- | --- | --- | --- |
| Sensory parameters | Leccino' | 'Štorta' | Ascolana tenera' | 'Mata' | 'Istrska belica' |  |  |
| Salty | 3.54 ± 2.23 a | 4.25 ± 1.34 a | 4.19 ± 2.60 a | 5.91 ± 1.89 ab | 7.88 ± 1.51 b |  |  |
| Bitter | 5.09 ± 2.43 a | 4.65 ± 1.98 a | 5.5 ± 2.52 a | 6.08 ± 2.21 a | 4.59 ± 1.30 a |  |  |
| Acid | 2.55 ± 0.71 a | 3.93 ± 1.52 ab | 3.79 ± 1.48 ab | 4.66 ± 1.47 b | 4.75 ± 1.03 b |  |  |
| Hardness | 3.30 ± 2.66 a | 6.53 ± 1.92 b | 5.89 ± 1.68 ab | 5.28 ± 2.11 ab | 5.73 ± 1.67 ab |  |  |
| Fibrousness | 4.00 ± 1.57 a | 6.34 ± 2.79 a | 4.33 ± 2.61 a | 6.09 ± 1.56 a | 4.94 ± 1.60 a |  |  |
| Chrunchiness | 2.73 ± 1.68 b | 5.13 ± 1.70 ab | 7.00 ± 1.73 a | 4.89 ± 1.58 ab | 5.50 ± 1.93 a |  |  |
| The values are means ± SD. Different letters indicate statistical differences among cultivars for each taste parameter according to Tukey’s multiple range test (*p* ≤ 0.05). | | | | | | | |
